# Testing parasite ‘intimacy’: the whipworm *Trichuris muris* in the European house mouse hybrid zone

**DOI:** 10.1002/ece3.2022

**Published:** 2016-03-17

**Authors:** Wasimuddin, Josef Bryja, Alexis Ribas, Stuart J. E. Baird, Jaroslav Piálek, Joëlle Goüy de Bellocq

**Affiliations:** ^1^ Institute of Vertebrate Biology of the Czech Academy of SciencesBrnoCzech Republic; ^2^ Department of Botany and ZoologyFaculty of ScienceMasaryk UniversityBrnoCzech Republic; ^3^ Biodiversity Research GroupFaculty of ScienceUdon Thani Rajabhat UniversityUdon ThaniThailand

**Keywords:** Hybrid zones, *Mus musculus*, parasite life history traits, phylogeography, population structure

## Abstract

Host‐parasite interaction studies across hybrid zones often focus on host genetic variation, treating parasites as homogeneous. ‘Intimately’ associated hosts and parasites might be expected to show similar patterns of genetic structure. In the literature, factors such as no intermediate host and no free‐living stage have been proposed as ‘intimacy’ factors likely constraining parasites to closely follow the evolutionary history of their hosts. To test whether the whipworm, *Trichuris muris,* is intimately associated with its house mouse host, we studied its population genetics across the European house mouse hybrid zone (HMHZ) which has a strong central barrier to gene flow between mouse taxa. *T. muris* has a direct life cycle and nonmobile free stage: if these traits constrain the parasite to an intimate association with its host we expect a geographic break in the parasite genetic structure across the HMHZ. We genotyped 205 worms from 56 localities across the HMHZ and additionally *T. muris* collected from sympatric woodmice (*Apodemus* spp.) and allopatric murine species, using mt‐COX1, ITS1‐5.8S‐ITS2 rDNA and 10 microsatellites. We show four haplogroups of mt‐COX1 and three clear ITS1‐5.8S‐ITS2 clades in the HMHZ suggesting a complex demographic/phylogeographic history. Microsatellites show strong structure between groups of localities. However, no marker type shows a break across the HMHZ. Whipworms from *Apodemus* in the HMHZ cluster, and share mitochondrial haplotypes, with those from house mice. We conclude *Trichuris* should not be regarded as an ‘intimate’ parasite of the house mouse: while its life history might suggest intimacy, passage through alternate hosts is sufficiently common to erase signal of genetic structure associated with any particular host taxon.

## Introduction

It has been suggested that the short generation time, and therefore potential for rapid divergence, of parasites relative to their hosts means the genetic structure of ‘intimate’ parasites can be used to resolve recent divisions in host populations (review in Whiteman and Parker 2005; Nieberding and Olivieri 2007). No intermediate host and a lack of free‐living stage are suggested as ‘intimacy’ factors likely to constrain parasites to closely follow the demo‐ and phylo‐geographic history of their hosts (Nieberding and Olivieri 2007). In contrast, intermediate host(s), host generalism, presence of a free‐living phase, high potential for switching between host taxa, and high host vagility must be regarded as factors which would tend to break down intimate host–parasite associations. Using parasites to resolve host divisions requires an understanding of the balance between these opposing forces: barriers to the passage of parasites between host taxa versus mechanisms allowing such passage. Host hybrid zones are natural laboratories (Hewitt 1988) for understanding such balances, in particular tension zones (Barton and Hewitt 1985) which are maintained by a similar balance of forces: host dispersal versus selection against hybrid hosts producing barriers to host gene flow. However, studies of host‐parasite interactions across hybrid zones have largely focussed on host genetic variability, treating their parasites as homogeneous (Detwiler and Criscione 2010).

The first such study for an animal host (Sage et al. 1986) examined helminth load across the European house mouse hybrid zone (HMHZ) where eastern *Mus musculus musculus* (hereafter Mmm) and western *Mus musculus domesticus* (hereafter Mmd), after having diverged for around 500,000 years in isolation (Boursot et al. 1993; Geraldes et al. 2008; Duvaux et al. 2011), have come back into contact forming a narrow hybrid zone extending from Scandinavia in the north to the Black sea in the south (Boursot et al. 1993; Jones et al. 2010; Ďureje et al. 2012). Studies of helminth load across this zone (Sage et al. 1986; Moulia et al. 1993; Baird et al. 2012) have made no attempt to assess genetic structure of the parasites. Two recent studies have, however, investigated parasite genetic structure: for an apicomplexan protozoan, *Cryptosporidium tyzzeri* (Kváč et al. 2013) and a virus, the murine cytomegalovirus (MCMV, Goüy de Bellocq et al. 2015). In both cases, the genetic structure of the parasite showed a clear break across the HMHZ, with each mouse subspecies harboring genetically distinct pools of parasites. That is: although the hosts are in contact, and there is host gene flow, both the hosts and their parasites maintain distinct genetic signatures. With respect to the hosts, it has been suggested taxa maintaining genetic distinction in the face of such gene flow should be regarded as species (Mallet 2005). With regard to the parasites, it seems appropriate to describe taxa maintaining genetic distinction in the face of host taxon contact as intimate with those hosts.

From this point of view, host contact zones provide an objective measure of whether or not the balance of forces affecting parasite passage allows an intimate host–parasite relationship. By this measure, the nematode *Longistriata caudabullata* does not have an intimate host–parasite relationship with the short‐tailed shrews, *Blarina brevicauda*, and *B. hylophaga*, which form a hybrid zone in central North America: the parasites show no pattern of mitochondrial subdivision across the host contact, despite having no intermediate host, no mobile free‐living stage and hosts with low vagility (Brant and Ortí 2003). Conversely, generalist parasites such as ticks can show host race formation in sympatric host species (Mccoy et al. 2001). These observations would argue that the balance of forces affecting parasite passage is not well predicted by current considerations of intimacy factors, though detailed studies may improve how such factors are weighed.

Here, we carry out a detailed study of whipworm parasites across the HMHZ. The whipworm *Trichuris muris* (family: Trichuridae) is a gastrointestinal nematode parasite of murine rodents (Feliu et al. 2000). It has a direct life cycle with a nonmobile free‐living phase, the eggs in mouse feces taking about 2 months to embryonate and become infective (Cliffe and Grencis 2004). These characteristics might argue for an intimate host–parasite relationship under current criteria. *T. muris* is common in Mmd and Mmm mice in Europe (reviewed in Goüy de Bellocq et al. 2012) and has a prevalence of 21% in the HMHZ as a whole, but with a significantly lower parasite load in hybrids compared to parental house mice (Baird et al. 2012). The lower parasite load in the center of the zone together with the relative dependency of the parasite for dispersal on its host suggests that the HMHZ should act as a strong barrier to parasite passage. If this scenario is correct, and given the potential for fast divergence of short life cycle parasites, we should expect two distinct genetic pools of parasites on each side of the HMHZ.

However, given the abovementioned poor predictive power of similar parasite life history traits in the helminth/shrew system (Brant and Ortí 2003), we also explore an alternative hypothesis. Whipworms found in other murine species in Europe, for example woodmice (*Apodemus* sp.) and rats (*Rattus* sp.) are also identified as *T. muris* (Feliu et al. 2000; Callejón et al. 2010), suggesting the *T. muris* found in the house mouse may not be host‐specific. In a recent phylogeographic study of worms from murine hosts in Europe that were identified as *T. muris*, Callejón et al. (2010) suggested two distinct taxa exist in continental Europe, a western continental type (WCE) widespread from Northern Spain to Denmark and an eastern continental type (ECE) covering at least Croatia, Romania, and Turkey. While it is tempting to propose that the transition between the two *T. muris* types could coincide with the location of the HMHZ throughout Europe, only Western house mice (Mmd), found to carry the western *T. muris* type, were included in their study (all worms from the Balkan peninsula and Turkey were isolated from other murine hosts but not from the Eastern house mice; Mmm). Within each *T. muris* type, no genetic distinction according to host species was found. It should be noted, however, that the marker used in this study, the ITS1‐5.8S‐ITS2 region of the ribosomal DNA (rDNA), is highly conserved and may simply not be variable enough to pick‐up potential host race formation in sympatric hosts – this might require more polymorphic markers such as microsatellites (Mccoy et al. 2001). The data available in the literature are insufficient to determine which among the three genera *Mus*,* Rattus*, and *Apodemus*, is the preferential host of the potentially shared *T. muris*. In any of the given host species, the prevalence is highly varia‐ble according to the locality (e.g., in *Apodemus sylvati‐cus*, 0.9% < prevalence < 19.4%) (Behnke et al. 1999, 2009; Goüy de Bellocq et al. 2003); in any given locality, prevalence can differ markedly between hosts (e.g., Mmd and *Rattus rattus* in Sicily, Milazzo et al. 2003). If a singular *T. muris* taxon indeed parasitizes all murine rodents without any marked preference, we might then expect no match between the genetic variability of *T. muris* and house mouse genetic structure as parasite passage may be possible via the other hosts despite any barrier to passage between the house mouse taxa. Our second scenario is then that, despite scoring highly on intimacy criteria, the frequency of passage of *T. muris* through other hosts is sufficiently high to swamp any possibility of an intimate relation with the house mouse.

To test which of these two scenarios best fits *T. muris* in the HMHZ, we used a dataset composed of mitochondrial sequences and genotypes at ten polymorphic nuclear microsatellites. We also sequenced the ITS1‐5.8S‐ITS2 region of rDNA to compare how the haplotypes of the HMHZ fit with the *T. muris* phylogeographic pattern reported by Callejón et al. (2010). We addressed the following questions: (1) Is there a break in the population genetic structure of *T. muris* across the HMHZ? (2) Do the ITS1‐5.8S‐ITS2 rDNA lineages found in the HMHZ correspond to the western and eastern clades previously suggested across Europe; (3) Does *T. muris* show population structure at the murine species/locality level? (4) What is the inferred role of other sympatric murine host species in the transmission of *T. muris* across the HMHZ?

## Materials and Methods

### Sampling and DNA extraction

**Figure 1:**
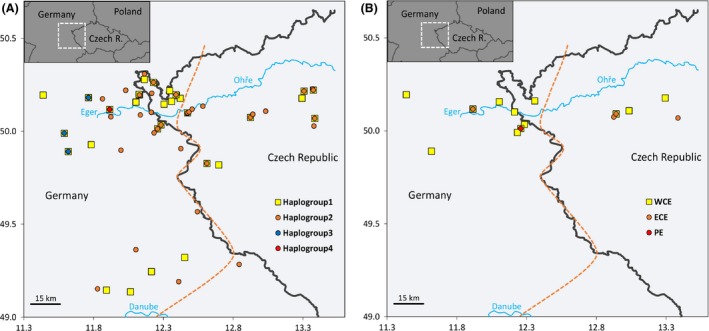
Map of sampled localities along with their corresponding (A) mitochondrial haplogroups (numbering of haplogroups corresponds to Fig. 2), (B) ITS1‐5.8S‐ITS2 clades (clades correspond to Fig. 4) of *Trichuris muris* (see the text for more details). Orange line represents consensus centre of host hybrid zone between western Mmd and eastern Mmm based on Ďureje et al. (2012).

*Trichuris muris* worms were collected from house mice at 56 localities across a transect (125 km long on South‐North axis and 140 km wide on Western‐Eastern axis) stretching from northeastern Bavaria (Germany) to western Bohemia (Czech Republic) between 2007 and 2013 (Fig. 1). All rodents were euthanized and dissected in a field laboratory. Worms were washed in sterile saline solution and preserved in 70% ethanol until morphological identification and DNA extraction. Additionally, *T. muris* individuals were collected from six woodmouse individuals (*Apodemus* spp.) from the same HMHZ area, from one Mmd captured in Northern Spain, and from one *Apodemus* sp. and one *Rattus rattus* from Southeastern France (Table S1). DNA was extracted from 214 worms, one nematode individual per host (except for six mice for which we extracted independently two nematode individuals per mouse) using the DNeasy^®^ DNA isolation kit (Qiagen, Hilden, Germany). DNA was eluted in 40 *μ*L of buffer AE. In general, DNA was extracted from male or immature *T. muris* worms. When no male or immature worms were available, we isolated the DNA from the upper part of a female body (stichosome) to avoid extracting the DNA from eggs contained in the posterior part of the body.

### Mouse genotyping

Host DNA was extracted from spleen and/or tail using DNeasy^®^ 96 Tissue Kit (Qiagen) following the manufacturer's instructions. All mice were genotyped either at 1401 ‘diagnostic’ X‐linked and autosomal SNP markers spaced approximately 1.86 Mb apart across the genome as part of a parallel study (see Wang et al. 2011) or at 11 diagnostic autosomal and X‐linked markers as described by Macholán et al. (2007). A Hybrid index (*HI*) was calculated to place any mouse on a linear scale from Mmd (*HI* = 0) to Mmm (*HI* = 1) depending on the count of *musculus* alleles at assayed loci (Macholán et al. 2007). Where it was necessary to categorize hosts, we divided them into western house mouse‐like and eastern house mouse‐like (Mmd: *HI *< 0.5 and Mmm: *HI *≥ 0.5, respectively).

### Mitochondrial and nuclear markers genotyping in whipworms

A portion of the mitochondrial cytochrome oxidase I (COX1) gene (1143 bp) was amplified using newly designed primers Trichuris_Cox1_F: CAGGAAATCACAAAAAAATTGG and Trichuris_Cox1_R: GAAAGTGTTGGGGYAKAAAAGTTA. The nuclear ITS1‐5.8S‐ITS2 rDNA region was amplified using NC2 and NC5 primers (Gasser and Hoste 1995). The PCRs were carried out in Mastercycler‐ep‐Gradient‐S (Eppendorf) and the mix contained 10 *μ*L of Multiplex PCR^™^ Kit (Qiagen), 0.5 *μ*mol/L of each primer, 2 *μ*L of DNA and double distilled water added to the final volume of 20 *μ*L. PCR started with an initial activation step at 95°C for 15 min, followed by 35 cycles of denaturation at 92°C (94°C for ITS region) for 60 sec, annealing at 51°C (52°C for ITS region) for 90 sec, extension at 72°C for 60 sec, and a final extension at 72°C for 10 min. PCR products were directly sequenced in both directions by VIB Genetic Service Facility (University of Antwerp, Belgium). We were aware that ITS1‐5.8S‐ITS2 rDNA may generate problematic chromatograms after sequencing because this marker is present in multiple potentially divergent copies in the genome. However, previous work on *T. muris* using cloning (Callejón et al. 2010) showed that the intraindividual variation is very low (often below 1%), and partly due to variation in repetition number of microsatellite motifs in the ITS1 and ITS2 regions which creates unreadable chromatograms. This problem does not affect all individuals in a study as per our previous experience (Ribas et al. 2013). We thus chose to perform direct sequencing on 95 randomly selected samples expecting that a subset of them could be directly genotyped without a more costly and time consuming cloning approach. Sequences were deposited in GenBank (Accession numbers KU575057‐KU575094).

### Microsatellite genotyping in whipworms

We first used three primer pairs originally designed for *Trichuris arvicolae* (Ta52, Ta87, Ta254; see for details, Deter et al. 2009), which crossamplified microsatellite loci in *T. muris*. Seven additional microsatellite loci were isolated from repeat‐containing contigs of the genome of *T. muris,* available at the Wellcome Trust Sanger Institute's depository (https://www.sanger.ac.uk/resources/downloads/helminths/trichuris-muris.html). For development of new microsatellite markers, we chose those contigs containing ideal repeats (continuous, with flanking regions of sufficient lengths). Primers were designed using the online tool Primer3Plus (http://primer3plus.com/cgi-bin/dev/primer3plus.cgi), and the forward primers were labeled fluorescently. Amplification success and polymorphism of the loci were initially tested on 10 samples from different localities (see more details in Table S1). A final set of 10 loci was amplified in three multiplex PCRs designed according to the dye and length of the products to avoid overlapping of loci with same labels (Table S2). The multiplex PCRs were carried out in a final volume of 10 *μ*L in a Mastercycler‐ep‐Gradient‐S (Eppendorf) with a mix containing 5 *μ*L of the Multiplex PCR^™^ Kit (Qiagen), 0.25 *μ*mol/L of each primer, 1 *μ*L of DNA and double distilled water. PCR started with an initial activation step at 95°C for 15 min, followed by 30 cycles with denaturation at 94°C for 60 sec, annealing at 58°C for 90 sec, extension at 72°C for 60 sec, and a final extension at 72°C for 10 min. Length of PCR products was analyzed on ABI3130xl Genetic Analyzer (Applied Biosystems, Carlsbad, CA, USA). The reaction mix for the fragment analysis composed of 0.4 *μ*L of Gene Scan‐500LIZ Size Standard (Applied Biosystems, Carlsbad, CA, USA), 12 *μ*L of formaldehyde, and 0.8 *μ*L of PCR product was denatured at 95°C for 5 min before capillary electrophoresis. Gene Mapper v3.7 (Applied Biosystems) was used for identification of peaks and detection of allele lengths.

### Data analysis

From 214 sampled whipworms, we obtained 195 COX1 sequences of 1143 bp including sequences of six worms from woodmice from the HMHZ and three worms, one each from woodmouse, black rat and Mmd from southern Europe (19 sequences from the HMHZ were removed from analysis due to poor sequence quality; see Table S1 for more details). A haplotype data file was generated and haplotype diversity (*h*) and nucleotide diversity (∏) were calculated in DnaSP v5 (Librado and Rozas 2009) and *p*‐distance between haplotypes were calculated in Mega 6.06 (Tamura et al. 2013). A median‐joining haplotype network was generated in Network 4.6 (Bandelt et al. 1999).

In total, 39 (out of 95 sequenced samples) sequences of the nuclear ITS1‐5.8S‐ITS2 rDNA region (969 to 976 bp long) could be read from direct sequencing. Among these 39, when the chromatogram of an individual appeared to have divergent copies of the marker, i.e., several peaks starting to appear on the chromatogram at the microsatellite motif of the ITS1 and/or ITS2 regions, one copy was much stronger and could easily be distinguished from the other(s) which appeared as very low baseline noise. We thus report here the ‘strong’ sequences of these 39 individuals. Sequences were aligned in Geneious 7.1.7 (Biomatters, Auckland, NZ) with the ITS1‐5.8S‐ITS2 rDNA sequences of *T. muris* of Murinae from continental Europe and Balearic Islands (Callejón et al. 2010) available in GenBank. *T. mastomysi* ITS1‐5.8S‐ITS2 rDNA sequence (Ribas et al. 2013) was used as outgroup. *T. mastomysi* is closer to *T. muris* than the outgroups of Callejón et al. (2010) making resolution of phylogenetic relationships between European clades more powerful.

Bayesian inference of phylogenetic relationships among mitochondrial and nuclear sequences was performed in MrBayes_v3.2.0 (Ronquist and Huelsenbeck 2003) in two independent runs, running each for 1,000,000 generations. The substitution model was chosen on the basis of Bayesian Information Criterion (BIC) in jModeltest 2.1.4 (Darriba et al. 2012). We also performed Maximum‐Likelihood (ML) phylogeny estimation using PhyML 3.0 (Guindon and Gascuel 2003; Guindon et al. 2010) with branch support evaluated by bootstrap (1000 replicates).

A total of 206 *T. muris* worms were genotyped at 10 microsatellite loci (Table S2). All loci consistently amplified in all samples. To analyze the genetic structure at locality level, we performed population genetic analyses for localities with at least five genotyped individuals. We tested Hardy–Weinberg equilibrium using GENEPOP 4.3 (Raymond and Rousset 1995) and calculated observed (*H*_o_) and expected (*H*_e_) (Nei's unbiased estimator) heterozygosities and pairwise *F*_ST_ values in FSTAT 2.9.3 (Goudet 1995). Genetic isolation by geographical distance was inferred by the Mantel test in GenAlEx version 6.5 (Peakall and Smouse 2006). Pairwise geographical distances were derived from longitudinal and latitudinal positions in GenAlEx and *F*_ST_ was transformed to *F*_ST_ / (1 − *F*_ST_) as suggested by Rousset (1997).

To infer the population structure of *T. muris* from the HMHZ, a Bayesian clustering approach was used on the microsatellite dataset as implemented in STRUCTURE version 2.3.4 (Pritchard et al. 2000). We also included in this analysis the six *T. muris* from *Apodemus* individuals sampled in the HMHZ area to test genetic structure at host species level. The analysis was replicated 10 times for each value of *K* from 1 to 15 using 100,000 iterations burn‐in followed by 500,000 iterations sampling the posterior. We used CLUMP to match cluster labeling within *K* levels (Jakobsson and Rosenberg 2007). Mean values of log likelihood, L(*K*), were plotted using Structure Harvester (Evanno et al. 2005; Earl and vonHoldt 2012). Graphic display of the STRUCTURE results was generated using DISTRUCT (Rosenberg 2004) displaying mouse individuals ordered according to localities (in a locality individuals are arranged according to their *HI* in increasing order) and then localities ranked according to average *HI* of host individuals in that locality (from 0 toward 1). Additionally, one model‐independent exploratory approach, Discriminant Analysis of Principal Components (DAPC), was applied to the microsatellite dataset, using the *adegenet* package (Jombart 2008; Jombart et al. 2010) implemented in R (R Core Team 2011). This analysis was performed without a priori information about localities using the *find.clusters* function, which ran from *K* = 2 to increasing number of clusters *K* = 59. The optimal *K* to explain population structure was chosen based on a significant dip in the BIC with increasing *K*.

## Results

### Mitochondrial DNA variation and its distribution

**Figure 2:**
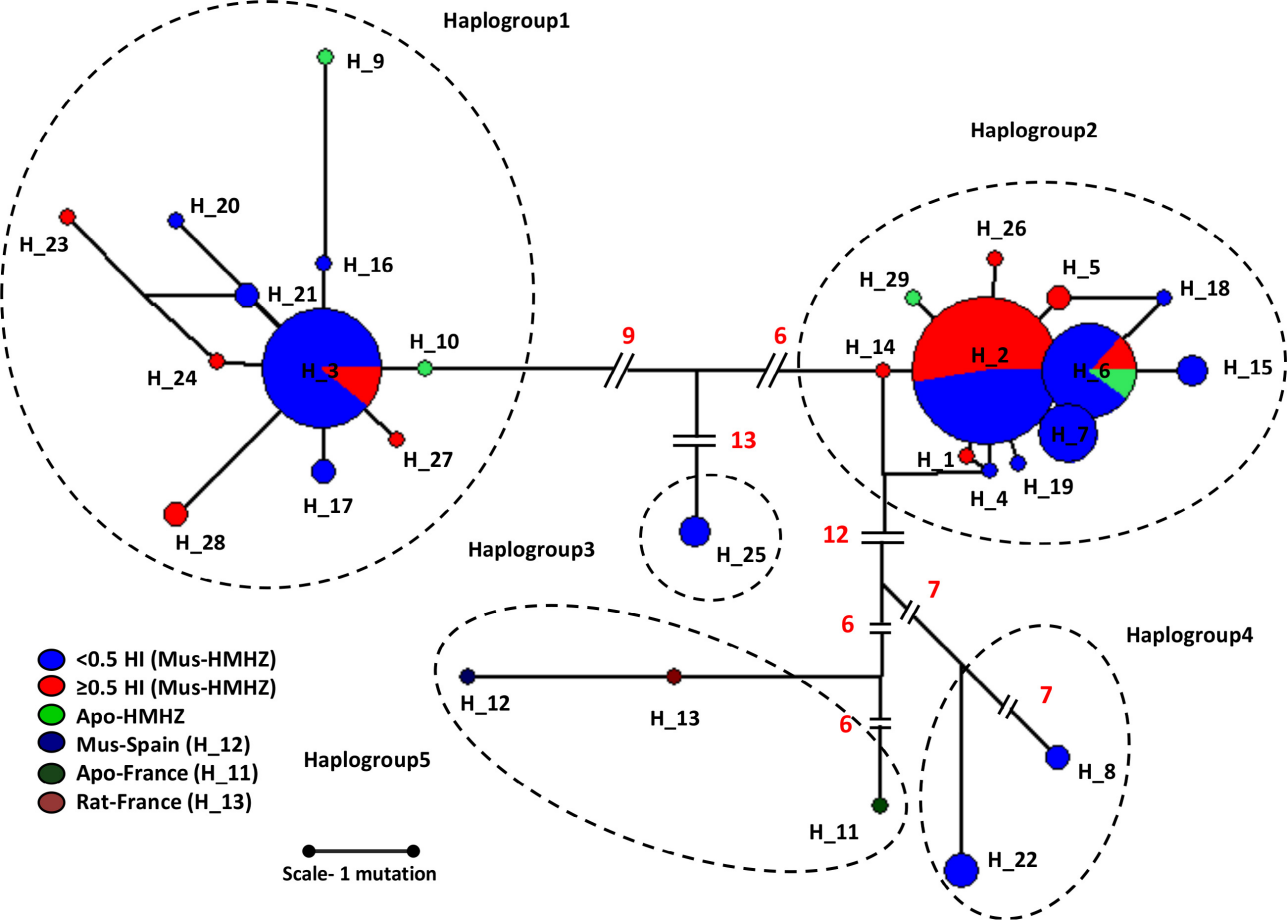
Median‐joining haplotype network of *Trichuris muris* mitochondrial partial COX1 sequences. Size of circles corresponds to the haplotype frequency and colors to genetic categories of hosts (Apo.: *T. muris* from *Apodemus* sp.; Mus: *T. muris* from *Mus musculus* spp., Rat: *T. muris* from *Rattus rattus*). Branch length represents mutational steps separating haplotypes. For simplicity, mutational steps only more than five are shown by red numbers on branch length. Haplotype numbers correspond to Table S1, haplogroups are shown by black dotted circles.

The mitochondrial dataset of 195 sequences contains 1062 invariable and 81 polymorphic sites with overall nucleotide diversity ∏ = 0.009. There were 29 distinct haplotypes, the total haplotype diversity (*h*) was 0.790. Maximum *p*‐distance between different haplotypes was 3.4%, minimum was 0.1% and average was 1.5%. Analyzing the pair of worms from the same host individual showed identical haplotypes (four pairs after remov‐ing two poor quality sequences) (Table S1). The mt‐haplotype network showed two big and three small haplogroups (Fig. 2). Representatives of four of these mt‐haplogroups occur in the HMHZ. Although two of the small haplogroups are found exclusively in Mmd‐like hosts (haplogroups 3 and 4; three and six individuals, respectively), the two large mt‐haplogroups which comprise the majority of the individuals are distributed across both Mmd‐ and Mmm‐like individuals (mt‐haplogroups 1 and 2; 59 and 124 individuals, respectively). In other words, the distribution of mt‐haplogroups 1 and 2 does not correspond to the genetic structure of the host hybrid zone (Fig. 1A). The three samples outside the HMHZ (southern Europe) made a distinct haplogroup (haplogroup 5) with each host species in southern Europe parasitized by a *T. muris* carrying a different mt‐haplotype (Fig. 2).

**Figure 3:**
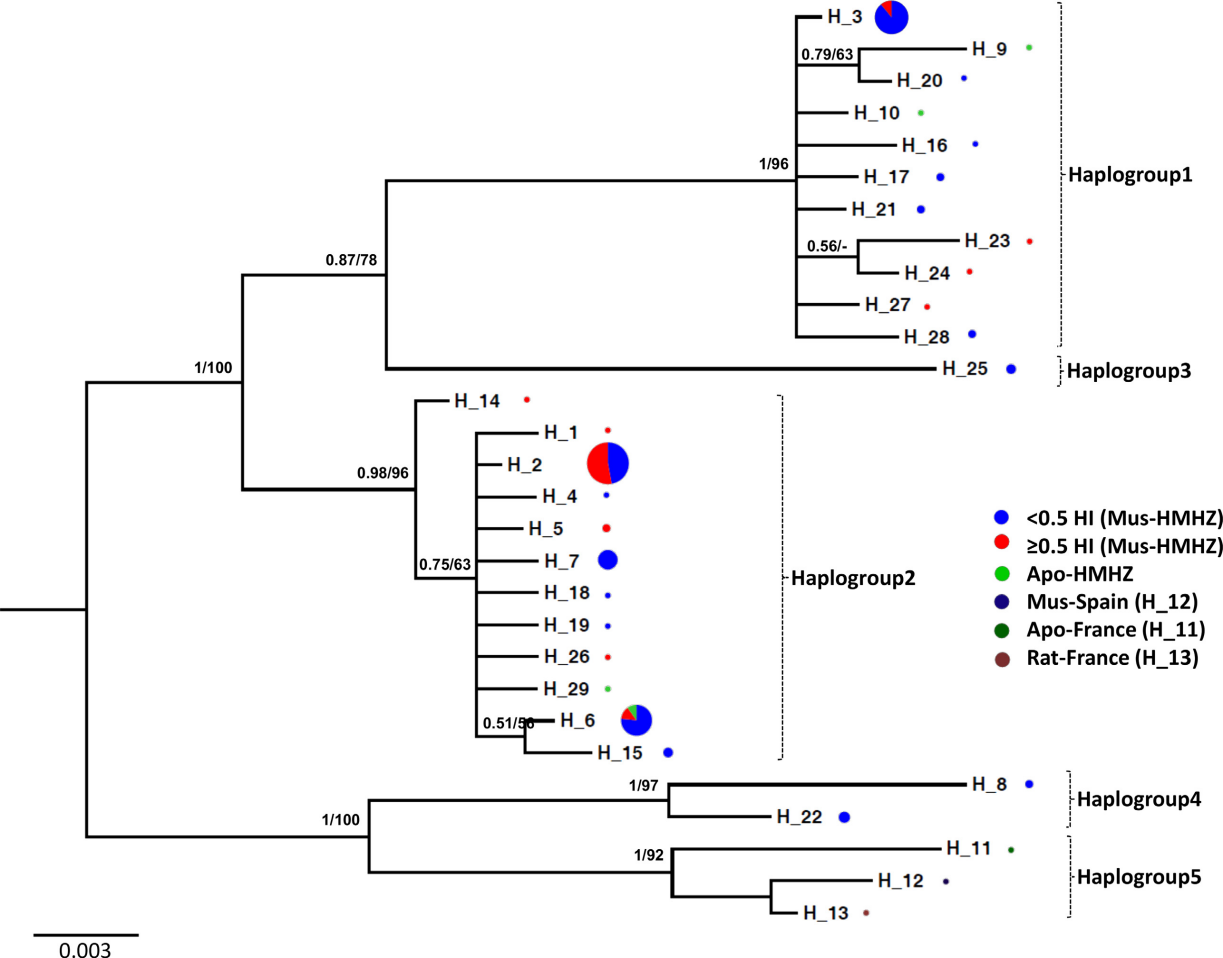
Bayesian tree (midpoint rooting) of *Trichuris muris* mitochondrial partial COX1 haplotypes (from H_1 to H_29) based on HKY+G model. Bayesian posterior probability values (>0.50) and ML bootstrap supports (>50%) are shown above particular nodes. Size of pie‐charts is proportionate to number of individuals and colors represent different genetic categories of hosts (Apo: *T. muris* from *Apodemus* sp.; Mus: *T. muris* from *Mus musculus* spp., Rat: *T. muris* from *Rattus rattus*). Haplotype numbers correspond to Table S1, haplogroups as defined in the network analysis are showed here by dotted line. The small scale bar represents the number of nucleotide substitutions per site.

jModeltest suggested the HKY+G substitution model for COX1 alignment with parameter values: ti/tv = 6.578, gamma shape = 0.074, base frequencies were A = 0.261, C = 0.169, G = 0.189, T = 0.379. The Bayesian mitochondrial phylogeny reconstruction provided support for two main lineages, each with internal structure (Fig. 3). The first lineage is composed of two sublineages: one was formed by three haplotypes carried by worms from Southern Europe (haplogroup 5), the second by haplotypes H_8 and H_22 from the Mmd side of the HMHZ (haplogroup 4). The second main lineage corresponds to haplogroup 1, 2, and 3 from the HMHZ. Within the HMHZ area, three woodmouse worms carried haplotype H_6 which was also found in worms from both Mmd and Mmm. The three other worms recovered from woodmice have unique haplotypes closely related to Mmd and Mmm worm haplotypes (Fig. 3). A similar tree topology was obtained with ML phylogenetic estimation (see ML bootstrap support in Fig. 3).

### Nuclear ITS1‐5.8S‐ITS2 rDNA diversity in the HMHZ and Europe

**Figure 4:**
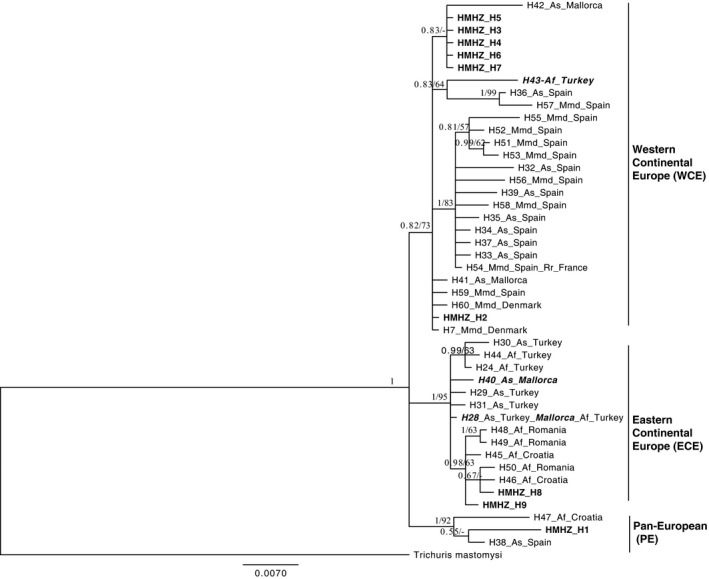
Phylogenetic tree estimated from Bayesian analysis of the ITS‐1, 5.8S, ITS‐2 region of *T. muris* individuals sampled in the house mouse hybrid zone and individuals sampled in house mice, rats, and woodmice across Europe and Mallorca island (Callejón et al. 2010). *Trichuris mastomysi* was used to root the tree. The two clusters defined by Callejón et al. (2010) are indicated on the right. Numbers above branches represent Bayesian posterior probability/ML bootstrap support (>50%). The small scale bar represents the number of nucleotide substitutions per site. The new haplotypes found in the house mouse hybrid zone (HMHZ) are indicated in bold and their numbers correspond to Table S1. For the haplotypes from Callejón et al. (2010), the haplotype number is given followed by the species in which it was found and the place/country. As: *Apodemus sylvaticus*, Af: *Apodemus flavicollis*, Mmd: *Mus musculus domesticus*, Rr: *Rattus rattus*. The haplotypes from Callejón et al. (2010) which are not belonging to the expected geographical cluster are highlighted in bold italics (e.g., while *H43_Af_Turkey* would be expected to belong to the Eastern clade, it is found in the Western continental clade defined by Callejón et al. 2010).

The ITS1‐5.8S‐ITS2 rDNA dataset of 39 sequences from HMHZ revealed nine different haplotypes. The variation between sequences was small and caused by different number of repeats of motif CTG in the ITS1 and GCA in the ITS2 regions and by few mutations present in both ITS regions, the 5.8S region being conserved between all haplotypes (except two mutations for haplotype HMHZ_H1). jModeltest suggested the HKY substitution model. The Bayesian phylogenetic reconstruction (Fig. 4) provided support for three clades: the first one (posterior probability = 0.82) corresponding to the “Western continental Europe (WCE)” clade of Callejón et al. (2010), which grouped together sequences of *T. muris* from Spain to Denmark, two haplotypes from Mallorca and one haplotype from Turkey. The majority of the samples from the HMHZ (33 individuals) is found in this clade and fall into six haplotypes (HMHZ_H2 to HMHZ_H7, see Table S1). The second clade (posterior probability = 1) corresponded to the “Eastern continental Europe (ECE)” clade of Callejón et al. (2010) which grouped together *T. muris* haplotypes from *A. sylvaticus* and *A. flavicollis* from Romania, Croatia, and Turkey and two *T. muris* haplotype from *A. sylvaticus* from Mallorca. Haplotypes HMHZ_H8 and HMHZ_H9 from five individuals in the HMHZ are found within this clade. The third clade (posterior probability = 1) grouped together haplotype HMHZ_H1 which belongs to an individual from the western side of the HMHZ with a *T. muris* haplotype from Croatia from *A. flavicollis* and a *T. muris* haplotype from Spain from *A. sylvaticus*. We thus call this the pan European (PE) clade. Plotting membership of the three ITS1‐5.8S‐ITS2 rDNA clades on a map, sequences of the first two clades (WCE and ECE) from *T. muris* in the HMHZ did not show any geographic break at the host contact zone (see Fig. 1B). It seems also that there is no correspondence between the two main COX1 haplogroups (haplogroup 1 and 2) and the WCE/ECE clades defined by the ITS1‐5.8S‐ITS2 rDNA region (Table S1) but more detailed analyses are barred due to the small sample sizes obtainable for the ITS1‐5.8S‐ITS2 rDNA marker.

### Genetic variation and structure assessed by nuclear microsatellites

All microsatellites were polymorphic, ranging from two to 11 alleles per locus (Table S2). There was no evidence of linkage disequilibrium (not shown), although many tests within locality samples were not possible because of locally fixed alleles. Worms from the same host individuals (six pairs) showed genotypes differing in average at five loci (range 3‐8). The average value per population for observed heterozygosity (*H*_o_) was 0.257 and expected heterozygosity (*H*_e_) was 0.354 (see Table S2 for heterozygosity values for each loci counted using populations with ≥5 individuals). Most populations showed significant deviations from Hardy–Weinberg equilibrium except HOHE1, OBIL, and WOHL (Table S3). Pairwise *F*_ST_ values between populations with ≥5 individuals ranged from 0.050 to 0.580 (Table S3) with the overall value of 0.271. No significant genetic isolation by geographical distance was observed between localities (Mantel test, *P* > 0.05) (Figure S1) suggesting important role of genetic drift (high genetic differences even between geographically close populations) and long‐distance migrations (low genetic differentiation between distant populations).

**Figure 5:**
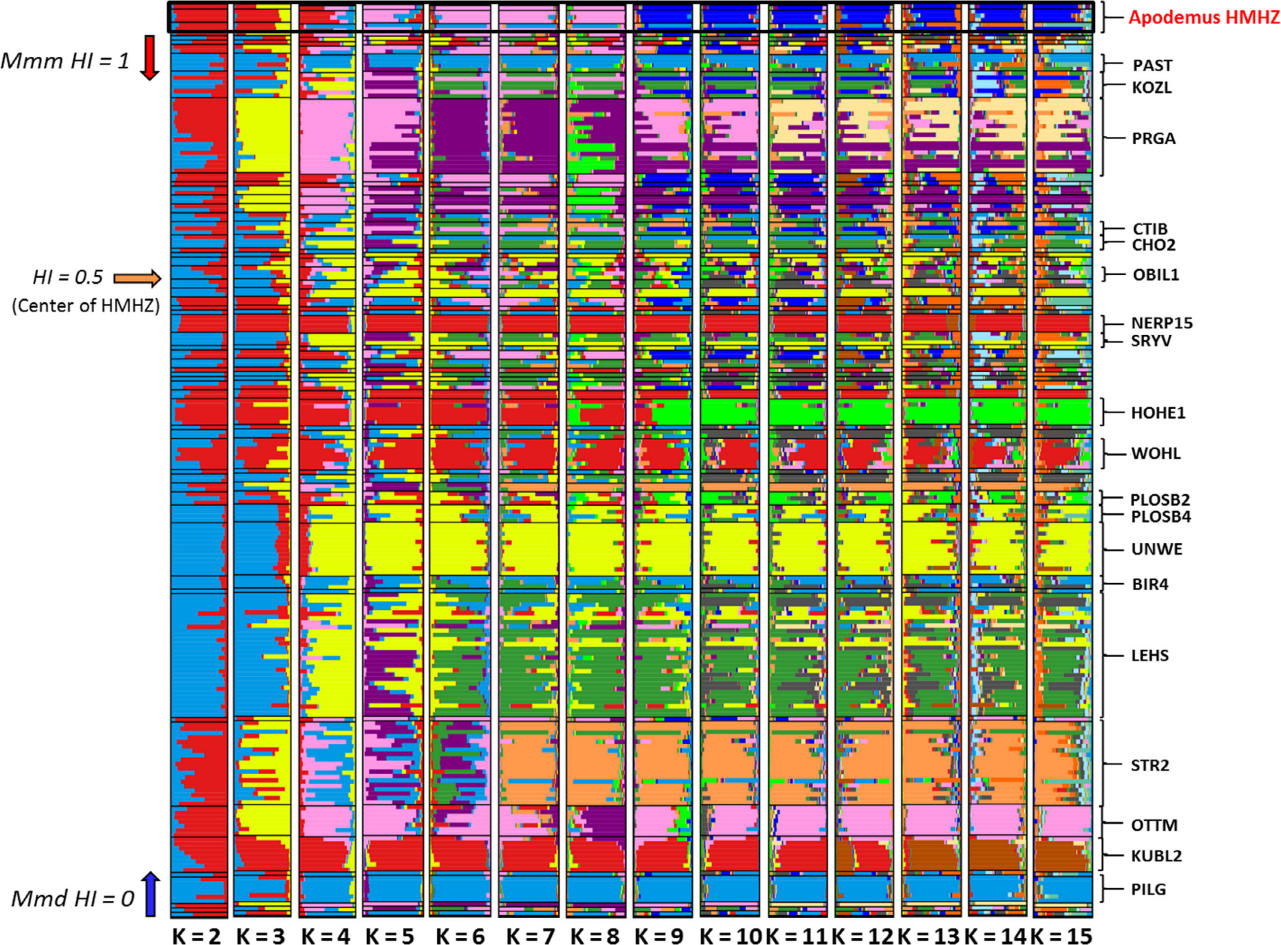
*Trichuris muris* cluster membership estimates in STRUCTURE models from *K* = 2 to *K* = 15. Individuals are ordered top to bottom according to their sampling localities as ranked by average host hybrid index. Results for six *Trichuris muris* from *Apodemus* spp. trapped in the HMHZ are placed at the top of the figure. Names of localities are shown on the right hand side (only localities with *N* ≥ 3 individuals are named), for more details on sampling localities see Table S1.

Likelihoods for STRUCTURE analyses for increasing numbers of genetic clusters (*K*) from 1 to 15, increased asymptotically (Figure S2) and the resulting estimates of cluster membership are shown in Figure 5. The cluster estimates correspond roughly to groups of localities subdivided to different levels depending on *K* and without respect to the host hybrid index (*HI*): Individuals from localities with similar host *HI* such as PILG and OTTM grouped in different clusters even at low *K* (see e.g., *K* = 2). Other localities such as PAST and PILG, despite being close to ‘pure’ Mmd and Mmm separated by 99 km cluster together even at high *K*. *T. muris* from *Apodemus* individuals caught in the HMHZ differ no more from house mouse *T. muris* taken from different localities, and never form an exclusive cluster.

**Figure 6:**
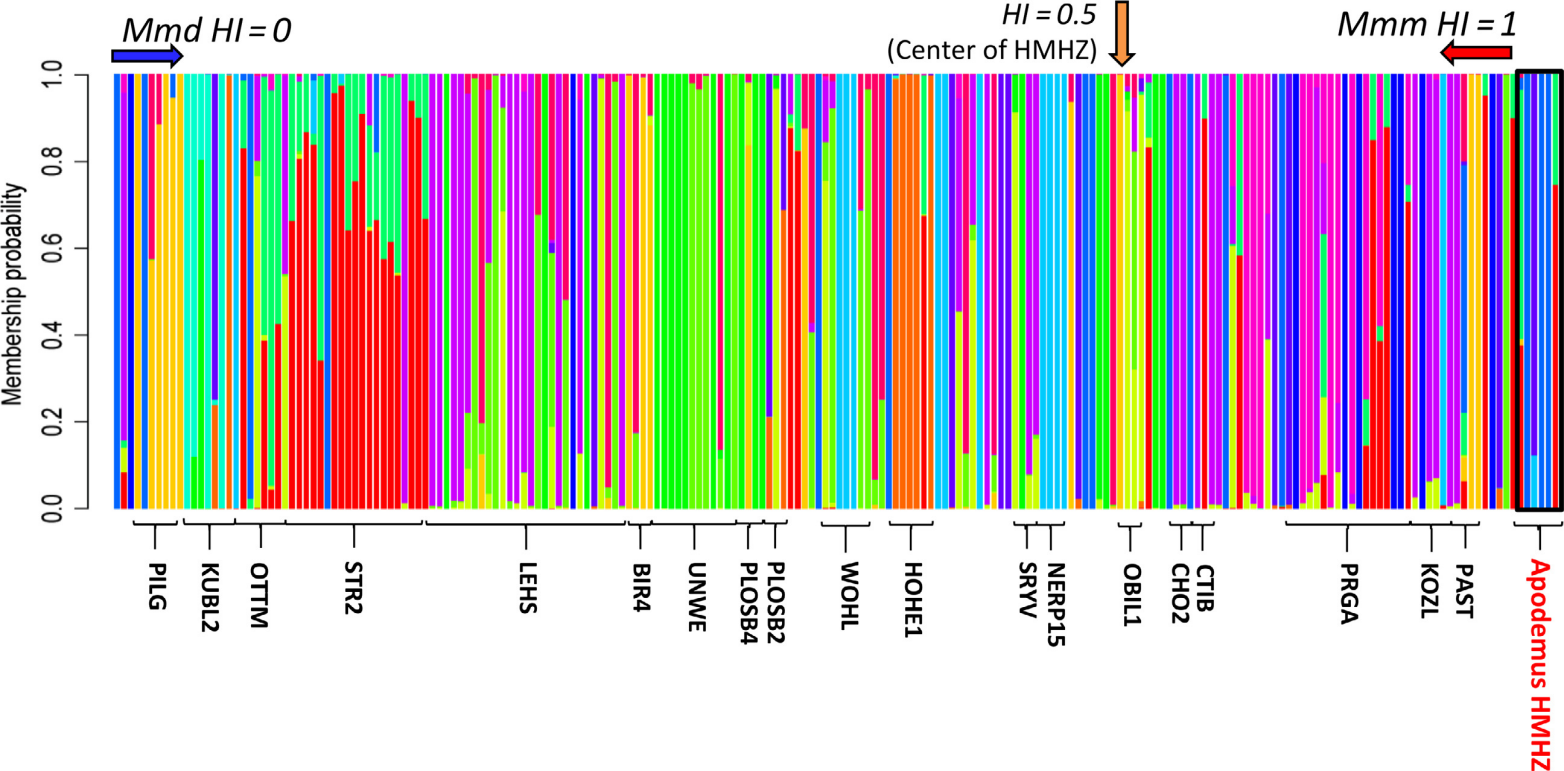
STRUCTURE like plot of DAPC analysis at *K* = 15. Each vertical line represents a *T. muris* genotype. These are ordered left to right by their sampling localities as ranked according to average host hybrid index. Names of localities are shown on bottom (only localities with *N* ≥ 3 individuals are named), for more details on sampling localities, see Table S1.

In DAPC analysis, running the *find.clusters* function, thirty five axes explained 100% of the total variance and over the range *K* = 1 to 59 the lowest BIC value was found at *K* = 15 (Figure S3). The first twenty PCA axes and five discriminant functions were retained and explained 93% of the total variance. As with the STRUCTURE analyses, clustering roughly corresponded to groups of localities (Fig. 6). Worms from localities with similar host *HI* grouped in different clusters. ‘Pure’ localities at the extremes of host *HI* share clusters of worms and *T. muris* from *Apodemus* individuals cluster with those from house mouse hosts.

## Discussion

We set out to compare two alternative hypotheses: whether (1) the characteristics of the host–parasite interaction between *Trichuris muris* and the house mouse nominally indicating an ‘intimate’ relationship (no intermediate host, a nonmobile free‐living phase) are predictive of the parasite's genetic structure across the European house mouse hybrid zone, or if, in contrast; (2) the frequency of *T. muris* passage through alternative hosts is sufficient to break any potential signal of intimate association with the house mouse host.

Our fine‐scale results for *T. muris* population genetics are consistent with previous studies. Pairs of *T. muris* from six mice had different nuclear, but similar mitochondrial, genotypes. This is consistent with accumulation of individual host worm load through repeated low‐level infection (Behnke and Wakelin 1973), the mean worm burden per mouse being 3.9 in the HMHZ (Baird et al. 2012). Within localities, there was a consistent deficit of heterozygotes at *T. muris* microsatellite loci suggesting local parasite bottlenecks and inbreeding consistent with the overdispersed nature of these parasites (Baird et al. 2012).

At a broader scale we show that, while there is plenty of signal of structure within our *T. muris* samples, it is at the level of sampling localities and without respect to the hybrid index of house mouse hosts. In addition, we show that *T. muris* from *Apodemus* spp., alternate hosts in the same sampling region are no more distinct from *T. muris* in the house mice than house mouse worms are when from different localities. From these two findings, we conclude hypothesis (2) is a better explanation of the data than hypothesis (1). That is: although *T. muris* seems to have a suite of traits making it a good candidate for an intimate host relationship, in nature it does not form an intimate relationship with house mice.

At the broadest phylogeographic scale using the ITS rDNA marker, we find two *T. muris* clades which correspond in part to the previously suggested WCE and ECE clades of Callejón et al. (2010) but also a third clade (pan‐European “PE”). The PE clade, including worms from Spain to Croatia, clusters together two haplotypes previously incorrectly included in the two Callejón et al. (2010) clades and a haplotype from the HMHZ. What evolutionary history of *T. muris* and its hosts would explain the observed pattern across Europe? Two obvious alternative sources of an east/west division are (1) *T. muris* carried by the two taxa of house mice diverged during a period in their history where passage in alternate hosts could not break their association – for example, as the two mice taxa spread in opposite directions round the Mediterranean and Black seas during their colonization of Europe (Boursot et al. 1993; Guénet and Bonhomme 2003). Once cohabiting the same region of Europe the *T. muris* in the house mouse taxa would start passage through shared alternate hosts (e.g., woodmice), breaking down the association between worm strains and host taxa such that eastern and western worm clades no longer strictly match eastern and western mouse clades. (2) The same scenario but *T. muris* is originally carried by two taxa of woodmice, which colonized Europe before the arrival of synanthropic house mice: *A. sylvaticus* and *A. flavicollis* are closely related woodmouse species which recolonized the Western Palearctic (and thus the HMHZ area) from different glacial refugia, *A. flavicollis* from the Italo‐Balkanic area and *A. sylvaticus* from the Iberian Peninsula (Michaux et al. 2005). Then, the later colonizing house mice are exposed to an existing east–west divergence in *T. muris* from established alternate hosts, loosing the relatively small number of distinctive *T. muris* strains which travelled with them. The third (PE) clade of *T. muris* may be a remnant of distinctive strains brought by either the house mouse or the rat, which colonized Europe even later (Colangelo et al. 2015). The scenario of house mouse acquisition of *T. muris* from resident woodmouse species is testable: *T. muris* in European house mice should be genetically closer to the worms from European *Apodemus* than to the worms found in house mice in the fertile crescent – the source of their European colonization. By the same argument, those fertile crescent house mouse‐*T. muris* may be genetically more similar to worms found in common fertile crescent alternate hosts.

Given the potential importance of parasites as biotic factors affecting evolutionary process, and the consequences of parasites moving between hosts (e.g., nematode parasites between humans and great apes, Hasegawa et al. 2014), it would be useful to be able to predict their host intimacy with better accuracy. We suggest greater care in the interpretation of the life history descriptions of parasites can aid predictive accuracy. In both the current case and that of the host–parasite relationship between the nematode *L. caudabullata* and shrews (Brant and Ortí 2003), the parasites are described as having no intermediate host, nonmobile free‐living stage and infecting hosts with low vagility. Although the free‐living stage is nonmobile, it may represent a considerable proportion of the duration of the entire parasite life cycle (2 months out of ~1 year (average host life expectancy) for *T. muris*, unknown for *L. caudabullata*), and thus a considerable life history investment by the parasite. An important question is: if a locality used by one host taxon (and therefore ‘infected’ with the free‐living stage of the parasite) lapses from host use, how often will it be repopulated (potentially by another host taxon) while ‘infection’ is still possible? If the duration of the free‐living stage of the parasite is long relative to this period of interhost‐taxon locality usage turnover then, that duration may be long enough to break host–parasite intimacy. Similar logic applies to descriptions of host as having ‘low vagility’: If host vagility is sufficient in the context of potential host taxa distribution (sympatric/parapatric) to allow interhost locality usage turnover faster than the free‐living stage of a parasite looses its infective potential then, that vagility may be sufficient to break intimacy.

How then can generalist parasites form host races when their hosts are sympatric (Mccoy et al. 2001)? If sympatric hosts are using the same physical locations (rather than exploiting separate locations/times within the same geography), interhost‐taxon parasite passage might break down, or prevent the development of, intimate host–parasite relationships. The host race forming ticks, parasitize seabirds that form mixed colonies, however it appears the birds nest at different times and on different substrates (Mccoy et al. 2001). Thus, sympatry per se is not a good indicator of the likelihood of interhost‐taxon passage of parasites. The three sympatric alternative hosts of *T. muris* in the present study (*A. sylvaticus*,* A. flavicollis*, and *R. norvegicus*) are judged to have partly overlapping ecological niches, but are rarely trapped simultaneously from the same farm building (Piálek, pers comm). This might suggest low passage rates between alternate hosts, but these same locations are almost always found to be occupied by at least one host (Piálek, pers comm), suggesting the period during which sites remain unoccupied is short, allowing parasite transmission through the free‐living stage in feces.

Our results are in strong contrast with two previous studies of parasite genetic structure in the HMHZ: for the protozoan *C. tyzzeri* and the murine cytomegalovirus (MCMV), each of which shows a break in genetic structure across the HMHZ (Kváč et al. 2013; Goüy de Bellocq et al. 2015). For the latter, there is also a qualitative difference in life history: the MCMV is thought to have only direct transmission via saliva – i.e., no free‐living stage. Direct transmission through saliva is likely within families of (allo‐grooming) conspecific mice and seems unlikely between more distantly related murine species (Poulin et al. 2006). This is therefore a very strong candidate for a host–parasite intimacy trait. The protozoan *C. tyzzeri* does however have a free‐living (encysted) stage, the oocyst, and has been found in a range of vertebrates (woodmice, voles, snakes, horses, including humans) (Kváč et al. 2013; Rašková et al. 2013; Wagnerová et al. 2015). It is possible that, given the apparently intimate relationship between *C. tyzzeri* and house mice, a further retrospective argument could be made for why its life history data would incorrectly predict its intimacy. However, this in some sense misses the point: if we can only reconstruct whether a parasite should be intimate or not from retrospective arguments then the power to *predict* whether a host–parasite relationship will be intimate is clearly low. Studies assuming host–parasite intimacy based only on life history traits should therefore be treated with caution.

## Conflict of interest

The authors declare no conflict of interest.

## Supporting information


**Figure S1.** Results of Mantel test showed non‐significant correlation between geographic (in km) and genetic (as *F*_ST_ / (1 − *F*_ST_)) distances among populations.
**Figure S2.** Log probability of data L(*K*) as a function of *K* (mean ± SD for 10 replicates).
**Figure S3.** Inference of the number of clusters in the DAPC analysis.

**Table S1.** Localities and their location, sample ID, host hybrid index (HI), host grouping based on hybrid index (Group), mitochondrial DNA haplotypes (mt hap), samples used in microsatellite analysis (Micro) and ITS1‐5.8S‐ITS2 haplotypes.
**Table S2.** Characteristics of 10 microsatellite loci and measures of genetic diversity (*N*_A_, number of alleles; *H*_o_, observed heterozygosity; *H*_e_, Nei's unbiased estimator of expected heterozygosity (mean values of heterozygosities were calculated using only population with ≥5 individuals))
**Table S3.** Hardy‐Weinberg equilibrium test (HWE; *P* values, FDR corrected significant *P*‐values are shown in bold) and pairwise *F*_ST_ values among populations (only populations with ≥5 individuals were used in these analyses).
