# Supplementary material for: Testing parasite ‘intimacy’: the whipworm Trichuris muris in the European house mouse hybrid zone
Source: Ecol Evol. 2016 Mar 17;6(9):2688–701. doi: 10.1002/ece3.2022 (PMC4798833; doi:10.1002/ece3.2022)

Figure S1. Results of Mantel test showed non-significant correlation between geographic (in km) and genetic (as $F_{ST}/(1-F_{ST})$) distances among populations.

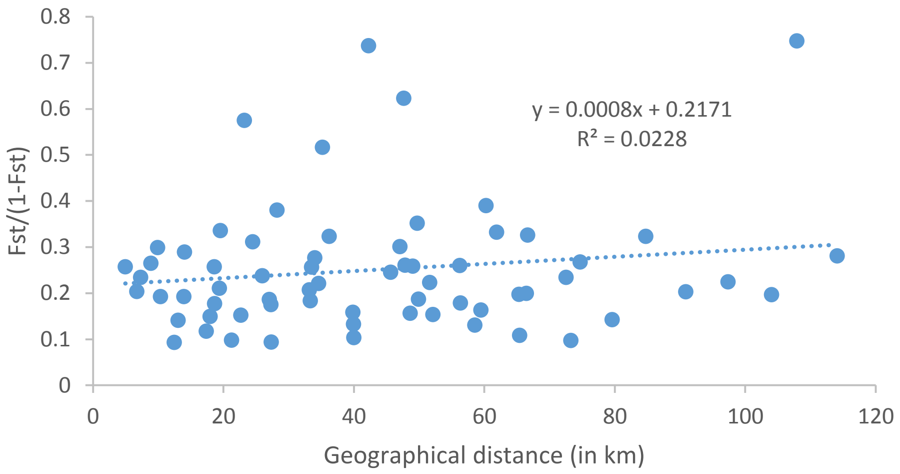

Figure S2. Log probability of data L(K) as a function of K (mean ± sd for10 replicates)

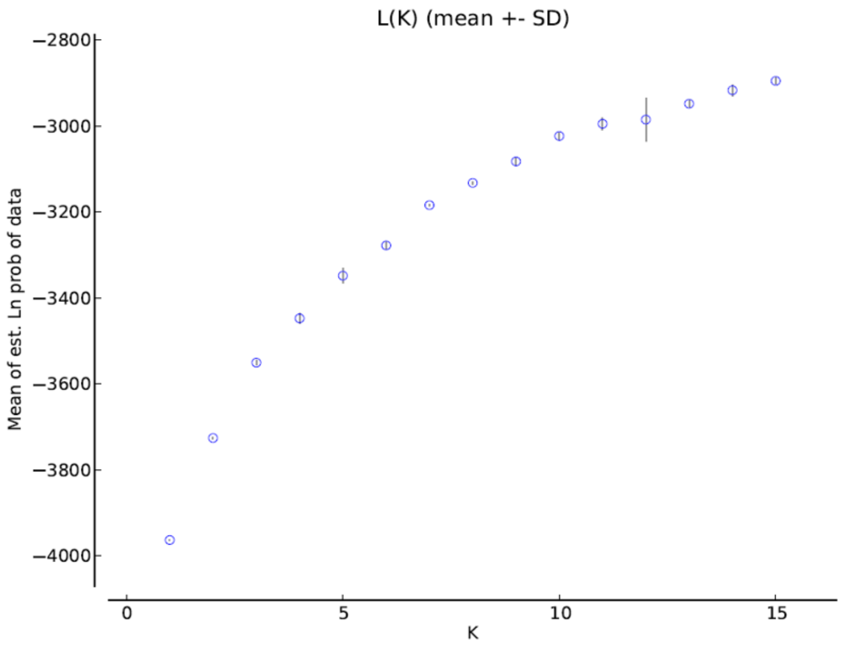

Figure S3. Inference of the number of clusters in the DAPC analysis. The lowest BIC value at K=15 (red circle) suggests best representation of the data.

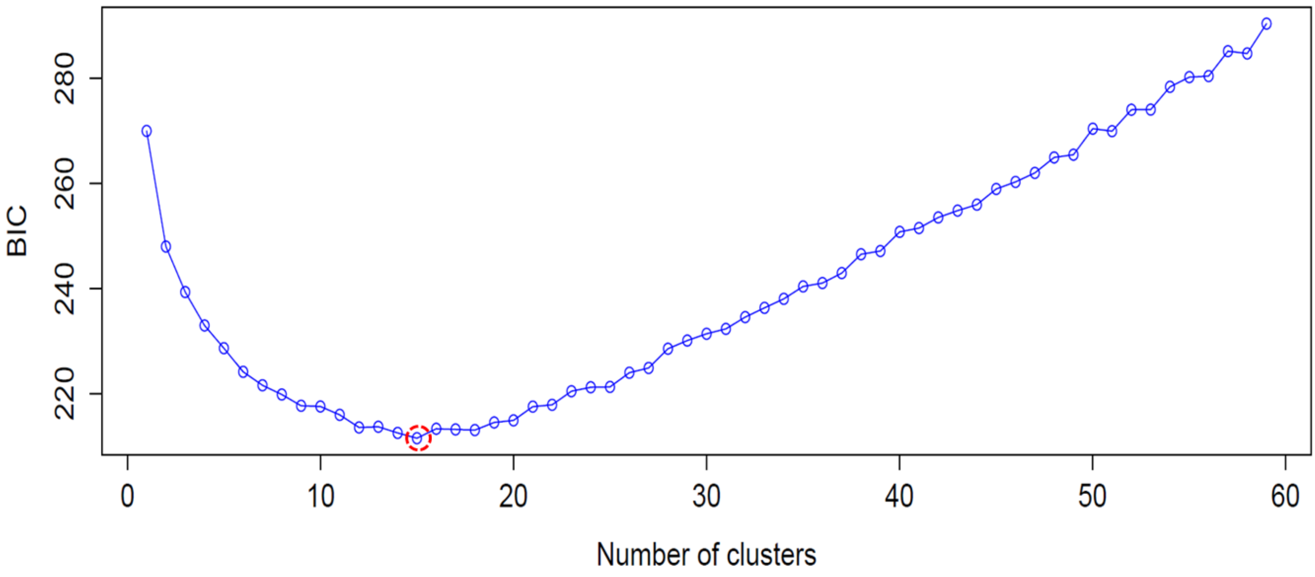

Supplement: Supplementary file 1 — Figure S1. Results of Mantel test showed non‐significant correlation between geographic (in km) and genetic (as F ST / (1 − F ST)) distances among populations. Figure S2. Log probability of data L(K) as a function of K (mean ± SD for 10 replicates). Figure S3. Inference of the number of clusters in the DAPC analysis. [file ECE3-6-2688-s001.pdf]
