# Supplementary material for: Testing parasite ‘intimacy’: the whipworm Trichuris muris in the European house mouse hybrid zone
Source: Ecol Evol. 2016 Mar 17;6(9):2688–701. doi: 10.1002/ece3.2022 (PMC4798833; doi:10.1002/ece3.2022)
Supplement: Supplementary file 2 — Table S1. Localities and their location, sample ID, host hybrid index (HI), host grouping based on hybrid index (Group), mitochondrial DNA haplotypes (mt hap), samples used in microsatellite analysis (Micro) and ITS1‐5.8S‐ITS2 haplotypes. Table S2. Characteristics of 10 microsatellite loci and measures of genetic diversity (N A, number of alleles; H o, observed heterozygosity; H e, Nei's unbiased estimator of expected heterozygosity (mean values of heterozygosities were calculated using only population with ≥5 individuals)) Table S3. Hardy‐Weinberg equilibrium test (HWE; P values, FDR corrected significant P‐values are shown in bold) and pairwise F ST values among populations (only populations with ≥5 individuals were used in these analyses). [file ECE3-6-2688-s002.docx]

**Table S1.** Localities and their location, sample ID, host hybrid index (HI), host grouping based on hybrid index (Group), mitochondrial DNA haplotypes (mt hap), samples used in microsatellite analysis (Micro) and ITS1-5.8S-ITS2 haplotypes.

| **Locality** | **Code** | **Latitude** | **Longitude** | **Mouse ID** | **HI** | **Group** | **mt hap** | **Micro** | **ITS1-5.8S-ITS2 hap** |
| --- | --- | --- | --- | --- | --- | --- | --- | --- | --- |
| Schrotzhofen | SCHR | 49° 9' 8" | 11° 49' 40" | SK2333 | 0.000 | <0.5 | H_6 | YES | NA |
| Röthelbach | ROTH | 49° 59' 22" | 11° 35' 23" | SK1303 | 0.000 | <0.5 | H_8 | YES | NA |
| Novosedly 177 | NOSED | 50° 16' 43" | 12° 9' 47" | SK1925 | 0.000 | <0.5 | H_3 | YES | NA |
| Pilgramsreuth | PILG | 50° 13' 14" | 12° 1' 47" | SK1870 | 0.000 | <0.5 | H_6 | YES | NA |
| Pilgramsreuth | PILG | 50° 13' 14" | 12° 1' 47" | SK1869 | 0.000 | <0.5 | H_2 | YES | NA |
| Pilgramsreuth | PILG | 50° 13' 14" | 12° 1' 47" | SK1871 ^†^ | 0.000 | <0.5 | H_2 | YES | NA |
| Pilgramsreuth | PILG | 50° 13' 14" | 12° 1' 47" | SK1861 | 0.000 | <0.5 | H_2 | YES | NA |
| Pilgramsreuth | PILG | 50° 13' 14" | 12° 1' 47" | SK1872 | 0.000 | <0.5 | H_2 | YES | NA |
| Pilgramsreuth | PILG | 50° 13' 14" | 12° 1' 47" | SK1865 | 0.000 | <0.5 | H_2 | YES | NA |
| Neuköslarn | NEUKO | 49° 53' 52" | 11° 59' 46" | SK1918 | 0.000 | <0.5 | H_2 | YES | NA |
| Kübelhof 2 | KUBL2 | 50° 11' 41" | 11° 26' 15" | SK1306 | 0.000 | <0.5 | NA | YES | NA |
| Kübelhof 2 | KUBL2 | 50° 11' 41" | 11° 26' 15" | SK1802 | 0.000 | <0.5 | H_17 | YES | NA |
| Kübelhof 2 | KUBL2 | 50° 11' 41" | 11° 26' 15" | SU4162 | 0.012 | <0.5 | H_3 | YES | NA |
| Kübelhof 2 | KUBL2 | 50° 11' 41" | 11° 26' 15" | SU4165 | 0.013 | <0.5 | H_3 | YES | NA |
| Kübelhof 2 | KUBL2 | 50° 11' 41" | 11° 26' 15" | SU4164 | 0.014 | <0.5 | H_3 | YES | NA |
| Kübelhof 2 | KUBL2 | 50° 11' 41" | 11° 26' 15" | SU4193 | 0.014 | <0.5 | H_3 | YES | NA |
| Kübelhof 2 | KUBL2 | 50° 11' 41" | 11° 26' 15" | SK1150 | 0.015 | <0.5 | H_17 | YES | HMHZ_H6 |
| Kübelhof 2 | KUBL2 | 50° 11' 41" | 11° 26' 15" | SK1166 | 0.016 | <0.5 | H_3 | YES | HMHZ_H6 |
| Ottmannsreuth | OTTM | 49° 53' 27" | 11° 37' 4" | SK1434 | 0.000 | <0.5 | H_6 | YES | HMHZ_H6 |
| Ottmannsreuth | OTTM | 49° 53' 27" | 11° 37' 4" | SK1120 | 0.014 | <0.5 | H_15 | YES | NA |
| Ottmannsreuth | OTTM | 49° 53' 27" | 11° 37' 4" | SK1078 | 0.015 | <0.5 | H_15 | YES | HMHZ_H6 |
| Ottmannsreuth | OTTM | 49° 53' 27" | 11° 37' 4" | SK1170 | 0.015 | <0.5 | NA | YES | NA |
| Ottmannsreuth | OTTM | 49° 53' 27" | 11° 37' 4" | SK1153 ^†^ | 0.016 | <0.5 | H_15 | YES | NA |
| Ottmannsreuth | OTTM | 49° 53' 27" | 11° 37' 4" | SK1079 | 0.017 | <0.5 | H_8 | YES | NA |
| Ottmannsreuth | OTTM | 49° 53' 27" | 11° 37' 4" | SK1168 | 0.019 | <0.5 | H_6 | YES | HMHZ_H6 |
| Straas 2 | STR2 | 50° 10' 53" | 11° 45' 45" | SK1361 | 0.000 | <0.5 | H_7 | YES | NA |
| Straas 2 | STR2 | 50° 10' 53" | 11° 45' 45" | SK1308 | 0.000 | <0.5 | H_7 | YES | NA |
| Straas 2 | STR2 | 50° 10' 53" | 11° 45' 45" | SK1293 | 0.000 | <0.5 | H_7 | YES | NA |
| Straas 2 | STR2 | 50° 10' 53" | 11° 45' 45" | ST9409 | 0.012 | <0.5 | NA | YES | NA |
| Straas 2 | STR2 | 50° 10' 53" | 11° 45' 45" | ST9394 | 0.017 | <0.5 | H_22 | YES | NA |
| Straas 2 | STR2 | 50° 10' 53" | 11° 45' 45" | SU4088 ^†^ | 0.017 | <0.5 | H_7 | YES | NA |
| Straas 2 | STR2 | 50° 10' 53" | 11° 45' 45" | SU1636 | 0.018 | <0.5 | H_7 | YES | NA |
| Straas 2 | STR2 | 50° 10' 53" | 11° 45' 45" | ST9375 | 0.019 | <0.5 | H_7 | YES | NA |
| Straas 2 | STR2 | 50° 10' 53" | 11° 45' 45" | SK1104 | 0.019 | <0.5 | H_7 | YES | NA |
| Straas 2 | STR2 | 50° 10' 53" | 11° 45' 45" | SU4134 | 0.020 | <0.5 | H_7 | YES | NA |
| Straas 2 | STR2 | 50° 10' 53" | 11° 45' 45" | SK1129 | 0.020 | <0.5 | H_7 | YES | NA |
| Straas 2 | STR2 | 50° 10' 53" | 11° 45' 45" | SU1659 | 0.020 | <0.5 | H_22 | YES | NA |
| Straas 2 | STR2 | 50° 10' 53" | 11° 45' 45" | SU4031 | 0.021 | <0.5 | H_7 | YES | NA |
| Straas 2 | STR2 | 50° 10' 53" | 11° 45' 45" | ST9410 ^*^ | 0.022 | <0.5 | H_22 | YES | NA |
| Straas 2 | STR2 | 50° 10' 53" | 11° 45' 45" | ST9410 ^*^ | 0.022 | <0.5 | NA | YES | NA |
| Straas 2 | STR2 | 50° 10' 53" | 11° 45' 45" | SU4167 | 0.022 | <0.5 | H_7 | YES | NA |
| Straas 2 | STR2 | 50° 10' 53" | 11° 45' 45" | ST9393 ^*^ | 0.022 | <0.5 | H_22 | YES | NA |
| Straas 2 | STR2 | 50° 10' 53" | 11° 45' 45" | ST9393 ^*^ | 0.022 | <0.5 | NA | YES | NA |
| Straas 2 | STR2 | 50° 10' 53" | 11° 45' 45" | SU4135 | 0.026 | <0.5 | H_7 | YES | NA |
| Eckartsreuth 1 | ECKR1 | 49° 55' 41" | 11° 46' 55" | SK1125 | 0.022 | <0.5 | H_16 | YES | NA |
| Lehsten | LEHS | 50° 7' 3" | 11° 54'53" | SK1160 | 0.018 | <0.5 | H_2 | YES | NA |
| Lehsten | LEHS | 50° 7' 3" | 11° 54'53" | ST9357 | 0.020 | <0.5 | H_2 | YES | NA |
| Lehsten | LEHS | 50° 7' 3" | 11° 54'53" | SU1491 | 0.021 | <0.5 | NA | YES | NA |
| Lehsten | LEHS | 50° 7' 3" | 11° 54'53" | SU4054 | 0.021 | <0.5 | H_25 | YES | NA |
| Lehsten | LEHS | 50° 7' 3" | 11° 54'53" | ST9355 | 0.021 | <0.5 | NA | YES | NA |
| Lehsten | LEHS | 50° 7' 3" | 11° 54'53" | SU4052 ^†^ | 0.023 | <0.5 | H_25 | YES | HMHZ_H7 |
| Lehsten | LEHS | 50° 7' 3" | 11° 54'53" | SU1427 | 0.023 | <0.5 | NA | YES | HMHZ_H8 |
| Lehsten | LEHS | 50° 7' 3" | 11° 54'53" | ST9377 ^*^ | 0.023 | <0.5 | H_2 | YES | NA |
| Lehsten | LEHS | 50° 7' 3" | 11° 54'53" | ST9377 ^*^ | 0.023 | <0.5 | H_2 | YES | NA |
| Lehsten | LEHS | 50° 7' 3" | 11° 54'53" | SU1506 | 0.024 | <0.5 | H_6 | YES | NA |
| Lehsten | LEHS | 50° 7' 3" | 11° 54'53" | SU4028 | 0.024 | <0.5 | H_2 | YES | HMHZ_H7 |
| Lehsten | LEHS | 50° 7' 3" | 11° 54'53" | SU4029 | 0.024 | <0.5 | H_2 | YES | NA |
| Lehsten | LEHS | 50° 7' 3" | 11° 54'53" | ST9390 | 0.024 | <0.5 | H_2 | YES | NA |
| Lehsten | LEHS | 50° 7' 3" | 11° 54'53" | SK1159 | 0.024 | <0.5 | H_2 | YES | NA |
| Lehsten | LEHS | 50° 7' 3" | 11° 54'53" | ST9405 ^*^ | 0.025 | <0.5 | H_2 | YES | NA |
| Lehsten | LEHS | 50° 7' 3" | 11° 54'53" | ST9405 ^*^ | 0.025 | <0.5 | H_2 | YES | NA |
| Lehsten | LEHS | 50° 7' 3" | 11° 54'53" | ST9370 | 0.025 | <0.5 | H_2 | YES | HMHZ_H8 |
| Lehsten | LEHS | 50° 7' 3" | 11° 54'53" | ST9356 | 0.025 | <0.5 | NA | YES | NA |
| Lehsten | LEHS | 50° 7' 3" | 11° 54'53" | SU4027 | 0.025 | <0.5 | H_25 | YES | HMHZ_H7 |
| Lehsten | LEHS | 50° 7' 3" | 11° 54'53" | SU4154 | 0.025 | <0.5 | H_2 | YES | HMHZ_H7 |
| Lehsten | LEHS | 50° 7' 3" | 11° 54'53" | ST9373 | 0.026 | <0.5 | NA | YES | NA |
| Lehsten | LEHS | 50° 7' 3" | 11° 54'53" | SU1426 | 0.026 | <0.5 | H_2 | YES | HMHZ_H4 |
| Lehsten | LEHS | 50° 7' 3" | 11° 54'53" | ST9406 | 0.026 | <0.5 | H_2 | YES | NA |
| Lehsten | LEHS | 50° 7' 3" | 11° 54'53" | ST9371 ^*^ | 0.027 | <0.5 | H_2 | YES | NA |
| Lehsten | LEHS | 50° 7' 3" | 11° 54'53" | ST9371 ^*^ | 0.027 | <0.5 | H_2 | YES | NA |
| Lehsten | LEHS | 50° 7' 3" | 11° 54'53" | ST9388 | 0.027 | <0.5 | H_2 | YES | NA |
| Lehsten | LEHS | 50° 7' 3" | 11° 54'53" | SU4064 | 0.029 | <0.5 | H_2 | YES | NA |
| Lehsten | LEHS | 50° 7' 3" | 11° 54'53" | ST9411 | 0.033 | <0.5 | NA | YES | NA |
| Benk 1 | BENK1 | 50° 10' 27" | 11° 51' 49" | SK1808 | 0.030 | <0.5 | H_6 | YES | NA |
| Birk 4 | BIR4 | 50° 4' 42" | 11° 55' 24" | SU4116 | 0.029 | <0.5 | H_2 | YES | NA |
| Birk 4 | BIR4 | 50° 4' 42" | 11° 55' 24" | SU4117 | 0.030 | <0.5 | H_6 | YES | NA |
| Birk 4 | BIR4 | 50° 4' 42" | 11° 55' 24" | SK1155 | 0.034 | <0.5 | H_6 | YES | NA |
| Unterweissenbach | UNWE | 50° 9' 25" | 12° 6' 12" | SK1824 | 0.000 | <0.5 | H_3 | YES | NA |
| Unterweissenbach | UNWE | 50° 9' 25" | 12° 6' 12" | SK1239 | 0.030 | <0.5 | H_3 | NA | NA |
| Unterweissenbach | UNWE | 50° 9' 25" | 12° 6' 12" | SK1248 | 0.030 | <0.5 | H_4 | NA | NA |
| Unterweissenbach | UNWE | 50° 9' 25" | 12° 6' 12" | SU1461 | 0.034 | <0.5 | H_3 | YES | NA |
| Unterweissenbach | UNWE | 50° 9' 25" | 12° 6' 12" | SU1604 | 0.035 | <0.5 | H_3 | YES | NA |
| Unterweissenbach | UNWE | 50° 9' 25" | 12° 6' 12" | SU1603 | 0.037 | <0.5 | H_3 | YES | NA |
| Unterweissenbach | UNWE | 50° 9' 25" | 12° 6' 12" | SU1432 | 0.039 | <0.5 | H_3 | YES | NA |
| Unterweissenbach | UNWE | 50° 9' 25" | 12° 6' 12" | SU1481 | 0.040 | <0.5 | H_3 | YES | NA |
| Unterweissenbach | UNWE | 50° 9' 25" | 12° 6' 12" | SU1602 | 0.040 | <0.5 | H_3 | YES | NA |
| Unterweissenbach | UNWE | 50° 9' 25" | 12° 6' 12" | SU1431 | 0.042 | <0.5 | H_3 | YES | HMHZ_H4 |
| Unterweissenbach | UNWE | 50° 9' 25" | 12° 6' 12" | SU1433 | 0.042 | <0.5 | H_3 | YES | NA |
| Unterweissenbach | UNWE | 50° 9' 25" | 12° 6' 12" | SU1429 ^†^ | 0.042 | <0.5 | NA | YES | NA |
| Unterweissenbach | UNWE | 50° 9' 25" | 12° 6' 12" | SU1482 | 0.045 | <0.5 | H_3 | YES | NA |
| Unterweissenbach | UNWE | 50° 9' 25" | 12° 6' 12" | SU1522 | 0.045 | <0.5 | H_3 | YES | HMHZ_H6 |
| Plössberg 42 | PLOSB4 | 50° 11' 43" | 12° 7' 42" | SK1842 | 0.050 | <0.5 | H_6 | YES | NA |
| Plössberg 42 | PLOSB4 | 50° 11' 43" | 12° 7' 42" | SK1838 | 0.050 | <0.5 | H_6 | YES | NA |
| Plössberg 42 | PLOSB4 | 50° 11' 43" | 12° 7' 42" | SK1816 | 0.050 | <0.5 | H_6 | YES | NA |
| Plössberg 42 | PLOSB4 | 50° 11' 43" | 12° 7' 42" | SK1812 | 0.050 | <0.5 | H_3 | YES | NA |
| Plössberg 25 | PLOSB2 | 50° 11' 54" | 12° 7' 31" | SK1837 | 0.050 | <0.5 | H_3 | YES | NA |
| Plössberg 25 | PLOSB2 | 50° 11' 54" | 12° 7' 31" | SK1813 | 0.050 | <0.5 | H_3 | YES | NA |
| Plössberg 25 | PLOSB2 | 50° 11' 54" | 12° 7' 31" | SK1814 | 0.050 | <0.5 | H_3 | YES | NA |
| Neuenreuth 8 | NEU8 | 50° 5' 16" | 12° 8' 4" | SU1544 | 0.049 | <0.5 | NA | YES | NA |
| Neuenreuth 8 | NEU8 | 50° 5' 16" | 12° 8' 4" | SU1486 | 0.052 | <0.5 | H_6 | YES | NA |
| Mokřiny | MOKR | 50° 12' 17" | 12° 12' 59" | SK1912 | 0.000 | <0.5 | H_6 | YES | NA |
| Mokřiny | MOKR | 50° 12' 17" | 12° 12' 59" | SK1901 | 0.125 | <0.5 | H_6 | YES | NA |
| Mýtinka 2 | MYT2 | 50° 8' 45" | 12° 18' 15" | SK1830 | 0.077 | <0.5 | H_3 | YES | NA |
| Wolfsbühl 4 | WOHL | 50° 0' 48" | 12° 15' 28" | SU1609 | 0.068 | <0.5 | H_6 | YES | NA |
| Wolfsbühl 4 | WOHL | 50° 0' 48" | 12° 15' 28" | SU1519 | 0.073 | <0.5 | H_3 | YES | NA |
| Wolfsbühl 4 | WOHL | 50° 0' 48" | 12° 15' 28" | SU1605 | 0.074 | <0.5 | NA | YES | HMHZ_H1 |
| Wolfsbühl 4 | WOHL | 50° 0' 48" | 12° 15' 28" | SK1095 ^†^ | 0.081 | <0.5 | H_3 | YES | HMHZ_H4 |
| Wolfsbühl 4 | WOHL | 50° 0' 48" | 12° 15' 28" | SU1610 | 0.082 | <0.5 | H_3 | YES | NA |
| Wolfsbühl 4 | WOHL | 50° 0' 48" | 12° 15' 28" | SK1272 | 0.104 | <0.5 | H_6 | YES | NA |
| Wolfsbühl 4 | WOHL | 50° 0' 48" | 12° 15' 28" | SK1273 | 0.113 | <0.5 | H_6 | YES | NA |
| Rosenbühl 2 | ROSE | 49° 59' 29" | 12° 13' 59" | SU1615 | 0.083 | <0.5 | H_6 | YES | HMHZ_H4 |
| Rosenbühl 2 | ROSE | 49° 59' 29" | 12° 13' 59" | SU1614 | 0.091 | <0.5 | H_6 | YES | HMHZ_H4 |
| Wiedenhof 1 | WHOF1 | 49° 8' 44'' | 11° 53' 31'' | SK2327 | 0.090 | <0.5 | H_20 | YES | NA |
| Hohenberg 1 | HOHBG1 | 50° 6' 7" | 12° 12' 46" | SU1530 | 0.085 | <0.5 | H_2 | YES | HMHZ_H4 |
| Hohenberg 1 | HOHBG1 | 50° 6' 7" | 12° 12' 46" | SU1656 | 0.097 | <0.5 | H_2 | YES | NA |
| Hohenberg 1 | HOHBG1 | 50° 6' 7" | 12° 12' 46" | SK1111 | 0.098 | <0.5 | H_2 | YES | HMHZ_H4 |
| Hohenberg 1 | HOHBG1 | 50° 6' 7" | 12° 12' 46" | SK1101 | 0.098 | <0.5 | H_2 | YES | HMHZ_H6 |
| Hohenberg 1 | HOHBG1 | 50° 6' 7" | 12° 12' 46" | SU1528 | 0.099 | <0.5 | NA | YES | HMHZ_H2 |
| Hohenberg 1 | HOHBG1 | 50° 6' 7" | 12° 12' 46" | SU4024 | 0.109 | <0.5 | H_2 | YES | HMHZ_H4 |
| Münchenreuth 31 | MUNR | 50° 2' 5" | 12° 16' 59" | SU1524 | 0.108 | <0.5 | H_3 | YES | NA |
| Münchenreuth 31 | MUNR | 50° 2' 5" | 12° 16' 59" | SU1640 | 0.125 | <0.5 | H_6 | YES | HMHZ_H3 |
| Křižovatka 79 | KRIZ79 | 50° 11' 48" | 12° 23' 22" | SK1911 | 0.120 | <0.5 | H_3 | YES | NA |
| Křižovatka 79 | KRIZ79 | 50° 11' 48" | 12° 23' 22" | SK1907 | 0.120 | <0.5 | H_19 | YES | NA |
| Hranice 787 | HRAN7 | 50° 18' 33" | 12° 9' 50" | SK1906 | 0.120 | <0.5 | H_6 | YES | NA |
| Haslarn | HASL | 49° 19' 16" | 12° 27'8" | SK2368 | 0.120 | <0.5 | H_3 | YES | NA |
| Pilmersreuth am Wald | PILW | 49° 54' 24" | 12° 25' 30" | SK1186 | 0.139 | <0.5 | H_6 | YES | NA |
| Irlaching 9 | IRLA9 | 49° 21' 46" | 12° 6' 9" | SK2337 | 0.120 | <0.5 | H_2 | YES | NA |
| Irlaching 9 | IRLA9 | 49° 21' 46" | 12° 6' 9" | SK2361 | 0.180 | <0.5 | H_2 | YES | NA |
| Kopaniny | KOPY | 50° 15' 46" | 12° 13' 42" | SK1893 | 0.160 | <0.5 | H_18 | YES | NA |
| Kopaniny | KOPY | 50° 15' 46" | 12° 13' 42" | SK1890 | 0.160 | <0.5 | H_3 | YES | NA |
| Křižovatka 2 | KRIZ2 | 50° 11' 33" | 12° 23' 37" | SK1124 | 0.174 | <0.5 | H_3 | YES | NA |
| Starý Rybník Vepřín | SRYV | 50° 9' 42" | 12° 21' 27" | SK1051 | 0.203 | <0.5 | H_3 | YES | HMHZ_H4 |
| Starý Rybník Vepřín | SRYV | 50° 9' 42" | 12° 21' 27" | SK1053 | 0.203 | <0.5 | H_3 | YES | NA |
| Starý Rybník Vepřín | SRYV | 50° 9' 42" | 12° 21' 27" | SK1054 ^†^ | 0.212 | <0.5 | H_3 | YES | NA |
| Nerping 15 | NER15 | 49° 14' 40" | 12° 12' 50" | SK2345 | 0.180 | <0.5 | H_3 | YES | NA |
| Nerping 15 | NER15 | 49° 14' 40" | 12° 12' 50" | SK2364 | 0.220 | <0.5 | H_3 | YES | NA |
| Nerping 15 | NER15 | 49° 14' 40" | 12° 12' 50" | SK2363 | 0.230 | <0.5 | H_3 | YES | NA |
| Nerping 15 | NER15 | 49° 14' 40" | 12° 12' 50" | SK2360 | 0.230 | <0.5 | H_3 | YES | NA |
| Plesná 275 | PLES2 | 50° 13' 7" | 12° 20' 40" | SK1908 | 0.230 | <0.5 | H_3 | YES | NA |
| Kreuth 4a | KREU4a | 49° 34' 1" | 12° 32' 36" | SK2312 | 0.230 | <0.5 | H_6 | YES | NA |
| Eichlberg 3 | EICHL | 49° 8' 10" | 12° 3' 51" | SK2351 | 0.190 | <0.5 | H_21 | YES | NA |
| Eichlberg 3 | EICHL | 49° 8' 10" | 12° 3' 51" | SK2357 | 0.270 | <0.5 | H_21 | YES | NA |
| Nová Ves 1 (CH) | NVCH1 | 50° 10' 39" | 12° 25' 14" | SK1142 | 0.173 | <0.5 | H_3 | YES | NA |
| Nová Ves 1 (CH) | NVCH1 | 50° 10' 39" | 12° 25' 14" | SK1151 | 0.295 | <0.5 | H_3 | YES | NA |
| Kirchenrohrbach | KIRBA | 49° 11' 28" | 12° 24' 34" | SK2321 | 0.440 | <0.5 | H_2 | YES | NA |
| Kirchenrohrbach | KIRBA | 49° 11' 28" | 12° 24' 34" | SK2319 | 0.500 | 0.5≤ | H_2 | YES | NA |
| Obilná 1 | OBIL1 | 50° 5' 58" | 12° 28' 21" | SU1419 | 0.711 | 0.5≤ | NA | YES | NA |
| Obilná 1 | OBIL1 | 50° 5' 58" | 12° 28' 21" | SU1451 | 0.737 | 0.5≤ | H_6 | YES | NA |
| Obilná 1 | OBIL1 | 50° 5' 58" | 12° 28' 21" | SU1415 | 0.753 | 0.5≤ | H_23 | YES | NA |
| Obilná 2 | OBIL2 | 50° 5' 47" | 12° 28' 17" | SK1249 | 0.725 | 0.5≤ | H_5 | YES | NA |
| Obilná 2 | OBIL2 | 50° 5' 47" | 12° 28' 17" | SU4180 | 0.778 | 0.5≤ | H_5 | YES | NA |
| Odrava 26 | ODR26 | 50° 6' 16" | 12° 29' 3" | SK1025 | 0.785 | 0.5≤ | H_6 | YES | NA |
| Grub 16, Furth | GRU16 | 49° 17' 1" | 12° 50' 34" | SK2320 | 0.860 | 0.5≤ | H_2 | YES | NA |
| Chotíkov 1 | CHOT1 | 50° 7' 9" | 12° 30' 21" | SU1599 | 0.792 | 0.5≤ | NA | YES | NA |
| Chotíkov 1 | CHOT1 | 50° 7' 9" | 12° 30' 21" | SU1498 ^†^ | 0.874 | 0.5≤ | H_2 | YES | NA |
| Chotíkov 1 | CHOT1 | 50° 7' 9" | 12° 30' 21" | SK1250 | 0.900 | 0.5≤ | H_2 | NA | NA |
| Chotíkov 1 | CHOT1 | 50° 7' 9" | 12° 30' 21" | SK1963 | 1.000 | 0.5≤ | H_6 | YES | NA |
| Ctiboř | CTIB | 49° 49' 36" | 12° 36' 41" | SK1849 | 0.923 | 0.5≤ | H_2 | YES | NA |
| Ctiboř | CTIB | 49° 49' 36" | 12° 36' 41" | SK1850 | 0.923 | 0.5≤ | H_2 | YES | NA |
| Ctiboř | CTIB | 49° 49' 36" | 12° 36' 41" | SK1831 | 0.923 | 0.5≤ | H_3 | YES | NA |
| Přehořov 11 | PRE11 | 50° 1' 41" | 13° 22' 41" | SK1213 | 0.950 | 0.5≤ | H_2 | YES | NA |
| Přehořov 11 | PRE11 | 50° 1' 41" | 13° 22' 41" | SK1210 | 0.955 | 0.5≤ | H_2 | YES | NA |
| Krásné Údolí | KRAU | 50° 4' 28" | 12° 55' 26" | SU1624 | 0.963 | 0.5≤ | H_2 | YES | NA |
| Krásné Údolí | KRAU | 50° 4' 28" | 12° 55' 26" | SU1622 | 0.969 | 0.5≤ | H_24 | YES | HMHZ_H8 |
| Přílezy - Pig house | PRPR | 50° 5' 28" | 12° 56' 24" | SU4059 | 0.971 | 0.5≤ | H_2 | YES | NA |
| Přílezy - Pig house | PRPR | 50° 5' 28" | 12° 56' 24" | SU4058 | 0.972 | 0.5≤ | H_2 | YES | HMHZ_H6 |
| Buškovice 2 | BUS2 | 50° 13' 18" | 13° 22' 27" | SU4110 | 0.971 | 0.5≤ | H_2 | YES | NA |
| Buškovice 2 | BUS2 | 50° 13' 18" | 13° 22' 27" | SU4112 | 0.972 | 0.5≤ | H_27 | YES | NA |
| Vrbička 23 | VRBI23 | 50° 10' 38" | 13° 17' 33" | SK1006 | 0.973 | 0.5≤ | H_3 | YES | HMHZ_H6 |
| Vrbička | VRBI | 50° 10' 40" | 13° 17' 34" | SU4171 | 0.973 | 0.5≤ | H_28 | YES | NA |
| Vrbička | VRBI | 50° 10' 40" | 13° 17' 34" | SU4169 | 0.974 | 0.5≤ | H_28 | YES | NA |
| Přílezy - Garage | PRGA | 50° 5' 29" | 12° 56' 27" | SU1629 | 0.967 | 0.5≤ | H_2 | YES | NA |
| Přílezy - Garage | PRGA | 50° 5' 29" | 12° 56' 27" | SU1626 ^†^ | 0.968 | 0.5≤ | H_2 | YES | NA |
| Přílezy - Garage | PRGA | 50° 5' 29" | 12° 56' 27" | SU1630 | 0.969 | 0.5≤ | H_2 | YES | HMHZ_H6 |
| Přílezy - Garage | PRGA | 50° 5' 29" | 12° 56' 27" | SU4055 | 0.970 | 0.5≤ | H_2 | YES | HMHZ_H6 |
| Přílezy - Garage | PRGA | 50° 5' 29" | 12° 56' 27" | SU4078 | 0.970 | 0.5≤ | H_2 | YES | NA |
| Přílezy - Garage | PRGA | 50° 5' 29" | 12° 56' 27" | SU1627 | 0.971 | 0.5≤ | H_2 | YES | NA |
| Přílezy - Garage | PRGA | 50° 5' 29" | 12° 56' 27" | SU1625 | 0.971 | 0.5≤ | H_2 | YES | HMHZ_H6 |
| Přílezy - Garage | PRGA | 50° 5' 29" | 12° 56' 27" | SU1628 | 0.972 | 0.5≤ | H_2 | YES | HMHZ_H8 |
| Přílezy - Garage | PRGA | 50° 5' 29" | 12° 56' 27" | ST9455 | 0.972 | 0.5≤ | H_2 | YES | HMHZ_H6 |
| Přílezy - Garage | PRGA | 50° 5' 29" | 12° 56' 27" | ST9492 | 0.972 | 0.5≤ | NA | YES | NA |
| Přílezy - Garage | PRGA | 50° 5' 29" | 12° 56' 27" | SU4075 | 0.973 | 0.5≤ | H_2 | YES | NA |
| Přílezy - Garage | PRGA | 50° 5' 29" | 12° 56' 27" | SU4076 | 0.974 | 0.5≤ | H_2 | YES | HMHZ_H6 |
| Přílezy - Garage | PRGA | 50° 5' 29" | 12° 56' 27" | SU4045 | 0.974 | 0.5≤ | H_2 | YES | NA |
| Přílezy - Garage | PRGA | 50° 5' 29" | 12° 56' 27" | SU4043 | 0.974 | 0.5≤ | H_26 | YES | HMHZ_H5 |
| Přílezy - Garage | PRGA | 50° 5' 29" | 12° 56' 27" | ST9444 | 0.976 | 0.5≤ | H_2 | YES | NA |
| Přílezy - Garage | PRGA | 50° 5' 29" | 12° 56' 27" | SK1214 | 1.000 | 0.5≤ | H_2 | YES | NA |
| Přílezy - Garage | PRGA | 50° 5' 29" | 12° 56' 27" | SK1215 | 1.000 | 0.5≤ | H_2 | YES | NA |
| Přílezy - Garage | PRGA | 50° 5' 29" | 12° 56' 27" | SK1241 | 0.970 | 0.5≤ | H_2 | NA | NA |
| Kozlov | KOZL | 50° 6' 30" | 13° 1' 57" | ST9400 ^*^ | 0.970 | 0.5≤ | H_2 | YES | NA |
| Kozlov | KOZL | 50° 6' 30" | 13° 1' 57" | ST9400 ^*^ | 0.970 | 0.5≤ | H_2 | YES | NA |
| Kozlov | KOZL | 50° 6' 30" | 13° 1' 57" | SK1040 | 0.971 | 0.5≤ | H_2 | YES | HMHZ_H6 |
| Kozlov | KOZL | 50° 6' 30" | 13° 1' 57" | SK1044 | 0.971 | 0.5≤ | H_2 | YES | HMHZ_H6 |
| Kozlov | KOZL | 50° 6' 30" | 13° 1' 57" | ST9401 | 0.976 | 0.5≤ | H_2 | YES | HMHZ_H6 |
| Kozlov | KOZL | 50° 6' 30" | 13° 1' 57" | SK1280 | 1.000 | 0.5≤ | H_2 | YES | NA |
| Kozlov | KOZL | 50° 6' 30" | 13° 1' 57" | SK1283 | 0.970 | 0.5≤ | H_2 | NA | NA |
| Pastuchovice | PAST | 50° 4' 9" | 13° 23' 3" | SK1133 | 0.968 | 0.5≤ | H_3 | YES | NA |
| Pastuchovice | PAST | 50° 4' 9" | 13° 23' 3" | SK1130 | 0.969 | 0.5≤ | NA | YES | HMHZ_H9 |
| Pastuchovice | PAST | 50° 4' 9" | 13° 23' 3" | SK1132 | 0.970 | 0.5≤ | H_6 | YES | NA |
| Pastuchovice | PAST | 50° 4' 9" | 13° 23' 3" | SK1207 | 1.000 | 0.5≤ | H_1 | YES | NA |
| Nepomyšl 172 | NEPO1 | 50° 12' 58" | 13° 18' 24" | SK1028 | 0.977 | 0.5≤ | H_3 | YES | NA |
| Nepomyšl 172 | NEPO1 | 50° 12' 58" | 13° 18' 24" | SK1026 | 0.979 | 0.5≤ | H_14 | YES | NA |
| Buškovice1 | BUS1 | 50° 13' 41" | 13° 22' 13" | ST9350 | 0.980 | 0.5≤ | H_2 | YES | NA |
| Šabina horses | SABIH | 50° 8' 6" | 12° 34' 58" | SK1216 ^†^ | 1.000 | 0.5≤ | H_2 | YES | NA |
| Lom u Tachova | LOMUT | 49° 49' 8" | 12° 41' 45" | SK1835 | 1.000 | 0.5≤ | H_3 | YES | NA |
| Lib | LIB | NA | NA | Apo 2441 | NA | NA | H_6 | YES | NA |
| Lib | LIB | NA | NA | Apo 2433 | NA | NA | H_29 | YES | NA |
| Schwetzendorf | SCHW | 49° 3' 50" | 12° 1' 40" | Apo 18 | NA | NA | H_6 | YES | NA |
| Schwetzendorf | SCHW | 49° 3' 50" | 12° 1' 40" | Apo 21 | NA | NA | H_6 | YES | NA |
| Schwetzendorf | SCHW | 49° 3' 50" | 12° 1' 40" | Apo 23 | NA | NA | H_9 | YES | NA |
| Kdyně | KDYNE | 49° 24' 13" | 13° 3' 28" | Apo 31 | NA | NA | H_10 | YES | NA |
| France-Pyrenees | FRANCE-P | 42° 29' 48" | 2° 21' 05" | Apo France | NA | NA | H_11 | NA | NA |
| Spain | SPAIN | 41° 37' 8" | 2° 17' 53" | *Mus* Spain | NA | NA | H_12 | NA | NA |
| France-Pyrenees | FRANCE-P | 42° 29' 48" | 2° 21' 5" | Rat France | NA | NA | H_13 | NA | NA |
|  |  |  |  |  |  |  |  |  |  |
| 205 whipworms from mouse-HMHZ from 56 localities | | | |  |  |  |  |  |  |
| 6 whipworms from *Apodemus*-HMHZ from 3 localities | | | | | | | | | |
| 3 whipworms from southern Europe (1 x *Mus,* 1 x *Apodemus,* 1 x *Rattus)* | | | | | |  |  |  |  |
| **In total 214 worms**  (Apo.: *T. muris* from *Apodemus* sp; Mus: *T. muris* from *Mus musculus sp.*, Rat: *T. muris* from *Rattus rattus*) | | | | | | | | |  |
| * (with same number) shows two whipworms from a host individual | | | | | |  |  |  |  |
| † sample used to test initial amplification of microsatellites | | | | | |  |  |  |  |
|  | |  | | | | | | | |

**Table S2.** Characteristics of 10 microsatellite loci and measures of genetic diversity {N_A_, number of alleles; Ho, observed heterozygosity; He, Nei’s unbiased estimator of expected heterozygosity (mean values of heterozygosities were calculated using only population with 5≤ individuals)}

| **Locus** | **Sequence** | **Repeat**  **motif** | **Dye*** | **Size (bp)** | **N_A_** | **Contig’s ID**  **(Sanger’s Database)** | **Ho/He** |
| --- | --- | --- | --- | --- | --- | --- | --- |
| Ta52 ^†^ | F:TAGGAGCGACGGTCATTAG | (GA)n | FAM^3^ | 328 | 4 | – | 0.137/0.158 |
|  | R:CAGATGAAGAGACGTTCGAC |  |  |  |  |  |  |
| Ta87 ^†^ | F:AACAGGAAGTGTCGCAGGTT | (GA)n | NED^3^ | 192 | 11 | – | 0.480/0.578 |
|  | R:CATATCCGGCGTCGTATACC |  |  |  |  |  |  |
| Ta254 ^†^ | F:CACTCTCCGCTTCTCTCGTT | (CA)n | HEX^3^ | 358 | 3 | – | 0.010/0.010 |
|  | R:AATAATGCCGCCGAAGAGAG |  |  |  |  |  |  |
| Tri_W_5 | F:CCGATATTCGTCTTCCGCTA | (GAC)n | NED^1^ | 150 | 9 | 00020.contig08140GAC8 | 0.582/0.543 |
|  | R:AAGATGCAGATCAAAGTGCG |  |  |  |  |  |  |
| Tri_W_7 | F:TCCACCAATCGACGAAAGAT | (TAG)n | VIC^1^ | 184 | 5 | 00118.contig04498TAG8 | 0.191/0.453 |
|  | R:GCAGTGGCAATCAGTCAATT |  |  |  |  |  |  |
| Tri_W_8 | F:GACGACGGTTCACAAAAAGT | (TGA)n | NED^2^ | 125 | 5 | 00053.contig07514TGA8 | 0.172/0.469 |
|  | R:AAGTCACACGCATGAAAGTG |  |  |  |  |  |  |
| Tri_W_10 | F:TTTGGGAGATAACATCCGCA | (TGC)n | PET^1^ | 110 | 5 | 00351.contig00847TGC9 | 0.514/0.538 |
|  | R:CTCATCCCAAAAACGCCAAT |  |  |  |  |  |  |
| Tri_W_14 | F:TTCTTACTGGGTGTGGTAGC | (TAC)n | VIC^2^ | 93 | 9 | 00082.contig02790TAC7 | 0.176/0.453 |
|  | R:CTTCACTTTGCTGATGCGAA |  |  |  |  |  |  |
| Tri_W_15 | F:ATCCAAACTGTCAGTGTCGT | (TCG)n | PET^2^ | 188 | 2 | 00040.contig08207TCG6 | 0.022/0.052 |
|  | R:ACAAGCGAAGTGGAAGTTTG |  |  |  |  |  |  |
| Tri_W_16 | F: CGTCGTGACACATTTCCATC | (GAC)n | FAM^2^ | 145 | 3 | contig11564GAC8 | 0.288/0.290 |
|  | R:TCGTCTGCTAATAGAGTCGC |  |  |  |  |  |  |

* Superscripts indicate the multiplex PCR set, in which the primer pair was used.

† Cross-amplified in *T. muris* (originally designed for *Trichuris arvicolae* by Deter *et al*. 2009)

Size (bp) - corresponds to the size of cloned product (Deter *et al*. 2009) or the size of fragments in the Sanger´s Database (<https://www.sanger.ac.uk/resources/downloads/helminths/trichuris-muris.html>).

**Table S3.** Hardy-Weinberg equilibrium test (HWE; p values, FDR corrected significant p-values are shown in bold) and pairwise *F*st values among populations (only populations with 5≤ individuals were used in these analyses).

| **Pop** | **HWE** | **FDR** | HOHE1 | KOZL | KUBL2 | LEHS | OBIL | OTTM | PILG | PLOSB | PRGA | STR2 | UNWE | WOHL |
| --- | --- | --- | --- | --- | --- | --- | --- | --- | --- | --- | --- | --- | --- | --- |
| HOHE1 | 0.5632 | 0.6144 | – |  |  |  |  |  |  |  |  |  |  |  |
| KOZL | 0.0022 | **0.0042** | 0.4677 | – |  |  |  |  |  |  |  |  |  |  |
| KUBL2 | 0.0210 | **0.0280** | 0.3780* | 0.2928 | – |  |  |  |  |  |  |  |  |  |
| LEHS | 0.0000 | **0.0000** | 0.4632* | 0.1898* | 0.2869* | – |  |  |  |  |  |  |  |  |
| OBIL | 0.8044 | 0.8044 | 0.3327 | 0.2307 | 0.2101 | 0.1428 | – |  |  |  |  |  |  |  |
| OTTM | 0.0019 | **0.0042** | 0.5130* | 0.3041 | 0.2949 | 0.3531* | 0.2960 | – |  |  |  |  |  |  |
| PILG | 0.0006 | **0.0018** | 0.5809 | 0.3245 | 0.3401* | 0.2525* | 0.2816 | 0.3184 | – |  |  |  |  |  |
| PLOSB | 0.0025 | **0.0042** | 0.2966 | 0.1875 | 0.1837* | 0.1372* | 0.0508 | 0.3357* | 0.2847 | – |  |  |  |  |
| PRGA | 0.0000 | **0.0000** | 0.3340* | 0.0910 | 0.3027* | 0.2569* | 0.1512* | 0.2283 | 0.3650* | 0.2117* | – |  |  |  |
| STR2 | 0.0154 | **0.0231** | 0.3490* | 0.2619* | 0.3017* | 0.2317* | 0.1281 | 0.2606* | 0.2969* | 0.2474* | 0.1980* | – |  |  |
| UNWE | 0.0000 | **0.0000** | 0.5513* | 0.3439 | 0.2771* | 0.1390* | 0.2036 | 0.4058* | 0.3876* | 0.1681* | 0.3663* | 0.3573* | – |  |
| WOHL | 0.0976 | 0.1171 | 0.3747* | 0.2189* | 0.2370* | 0.2192* | 0.1281 | 0.2630 | 0.3009* | 0.1714 | 0.2164* | 0.2010* | 0.2612* | – |

* Significant *Fst* values (according to adjusted p-values after bonferroni correction for multiple comparisons) tested after 1320 permutations.
